# Automating tumor–stroma ratio quantification in colon cancer patients from the UNITED study

**DOI:** 10.1016/j.esmoop.2025.105934

**Published:** 2025-12-30

**Authors:** F. Heilijgers, M. Polack, A.G.H. Roodvoets, E. Meershoek - Klein Kranenbarg, K.C.M.J. Peeters, R. Buettner, Y. Tolkach, W.E. Mesker, UNITED Collaboration, Floor Heilijgers, Gabi W. van Pelt, Koen C.M.J. Peeters, Marloes A. Smit, Meaghan Polack, Rob A.E.M. Tollenaar, Wilma E. Mesker, Elma Meershoek-Klein Kranenbarg, A.G.H. Roodvoets, Hein Putter, Augustinus S.L.P. Crobach, H. Gelderblom, G.R. Vink, Miriam Koopman, M.M. Lacle, L. Mekenkamp, Frédérique Beverdam, Jan Jansen, Maud Strous, Nicole W.J. Bulkmans, Joop van Baarlen, Ronald Hoekstra, Mark Sie, Natalja E. Leeuwis-Fedorovich, Koen A. Talsma, Emma Witteveen, M.P. Hendriks, Arash Bordbar, René Arensman, Valeska Terpstra, Arjan van Tilburg, M. Vermaas, J.W.T. Dekker, J. Han van Krieken, E.M.V. de Cuba, Mário Fontes-Sousa, A. Raimundo, Paula Borralho Nunes, João Cruz, R. Souza da Silva, Maria J. Brito, M. Cuatrecasas, María Teresa Rodrigo, Iván Archilla Sanz, Adriana Zucchiatti, S. Landolfi, S. Simonetti, S. Kjaer-Frifeldt, Jan Lindebjerg, Roger Feakins, Shonali Natu, Raed Al Dieri, Els Dequeker, Gordana Petrushevska, Pance Zdravkovski, Pance Karagjozov, Darko Dzambaz, Svetozar Antovic, Magdalena Bogdanovska Todorovska, Slavica Kostadinova Kunovska, Gorana Gasljevic

**Affiliations:** 1 Department of Surgery, Leiden University Medical Center, Leiden, The Netherlands; 2 Clinical Research Center, Department of Surgery, Leiden University Medical Center, Leiden, The Netherlands; 3 Institute of Pathology, University Hospital Cologne, Cologne, Germany

**Keywords:** colon cancer, tumor microenvironment, tumor–stroma ratio, disease-free survival, pathology, artificial intelligence

## Abstract

**Background:**

Colon cancer is a leading cause of cancer-related death worldwide, making accurate prognostic tools crucial. The tumor–stroma ratio (TSR) quantifies the proportion of stromal to tumor epithelial tissue and is a prognosticator in colon cancer. Stroma-high tumors are associated with worse outcomes and less benefit from adjuvant chemotherapy (ACT). Transitioning from visual to automated TSR scoring coincides with digital pathology advancements.

**Materials and methods:**

In this study, we validated a fully automated artificial intelligence-based TSR quantification algorithm in hematoxylin–eosin-stained slides from the UNITED cohort, including 853 stage II and III colon cancer patients who had not received neoadjuvant therapy. The algorithm segmented 11 tissue classes on whole-slide images and calculated TSR as stroma/(stroma + epithelial tumor). A 1-mm region of interest was implemented, and an optimal automated cut-off of 77% was derived using receiver operating characteristic analyses for disease-free survival (DFS) and overall survival (OS).

**Results:**

The median patient age was 67 years. Stroma-high patients had significantly worse DFS than stroma-low patients (3-year DFS 71% versus 82%, *P* < 0.001). This prognostic effect remained significant in the multivariate analysis (*P* = 0.016) and was consistent across stage II and III subgroups. Importantly, worse DFS was observed in both stroma-high stage II and III patients despite ACT (3-year DFS 74% versus 89%, *P* = 0.015; and 65% versus 79%, *P* = 0.002, respectively), suggesting potential predictive value. OS was worse in stroma-high patients as well (5-year OS 69% versus 85%, *P* = 0.002).

**Conclusion:**

We validated a clinically applicable tool for fully automated TSR quantification in colon cancer, including an optimal region of interest and cut-off value for automated scoring. We confirm the independent prognostic value of automated TSR for DFS and OS and highlight its influence on potential ACT benefit, underlining its clinical relevance. Our findings support its integration into digital pathology workflows.

## Highlights

• A clinically applicable tool for fully automated TSR quantification in colon cancer mirrors visually scoring accuracy.
• Automated TSR is an independent prognostic marker in stage II and III colon cancer.
• Stroma-high tumors (i.e. intratumoral stroma percentage >77%) are linked to significantly worse DFS and OS outcomes.
• TSR may predict benefit from adjuvant chemotherapy in stage II and III colon cancer.
• TSR is ready for integration into routine digital pathology workflows.

## Graphical abstract

**Figure:**
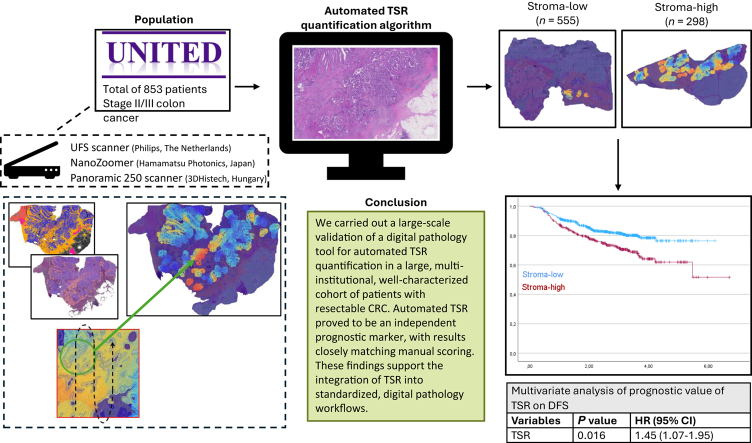

## Introduction

Colon cancer is among the most aggressive malignancies, ranking as the third leading cause of cancer-related deaths and accounting for ∼10% of all cancer cases.^1^ Consequently, colon specimens represent a substantial part of the daily diagnostic workload of pathologists. Recent advancements in digital pathology, particularly the integration of artificial intelligence (AI), have begun to transform the diagnostic landscape. AI-based computational tools, applied to digitized tissue sections, assist pathologists in routine tasks such as tumor detection, subtyping, and grading.^2^ These diagnostic tools, developed for various organs, have already demonstrated benefits, including enhanced prognostic value and reduced workload for pathologists.^3^, ^4^, ^5^

The tumor microenvironment, particularly the tumor stroma, has been shown to significantly impact patient outcomes. Research indicates that an abundance of tumor stroma correlates with worse outcomes, influencing tumor progression, metastasis, and resistance to therapies.^6^, ^7^, ^8^ A key histological parameter for evaluating this aspect of the tumor microenvironment is the tumor–stroma ratio (TSR), which quantifies the proportion of stroma compared with the tumor epithelial component. Our research group first developed the TSR in colon cancer, and subsequent studies have proven its prognostic value for almost all epithelial cancers.^9^, ^10^, ^11^ The UNITED study recently validated TSR as an independent prognosticator in colon cancer, confirming worse outcomes in stroma-high colon cancer patients.^12^ This significant result paved the way for TSR’s inclusion in the ninth edition of the TNM (tumor–node–metastasis) classification of Malignant Tumors by the Union for International Cancer Control and the American Joint Committee on Cancer.^13^ Furthermore, stroma-high colon cancer patients may experience chemotherapy resistance as they show less benefit from adjuvant chemotherapy (ACT), positioning TSR as not only a prognostic marker, but also a potential predictor of treatment response.^12^^,^^14^^,^^15^ This is in line with a need for de-escalation of current treatment regimens in an era where the number of patients will increase substantially, and treatment capacity will not keep up.

Currently, the assessment of the TSR is conducted by human observers who visually estimate the proportion by selecting the most invasive region of the tumor as determined for the T-category.^16^ Although previous studies in patients with colon cancer have demonstrated moderate to high interobserver agreement, with kappa values ranging from 0.68 to 0.97, variability remains, particularly around the critical 50% cut-off threshold.^6^^,^^9^^,^^17^ This underscores the susceptibility of the method to interobserver variability despite overall concordance.^18^ Especially for these cases, automating TSR quantification could be useful and facilitate its routine use in daily pathology practice.

Previous efforts to quantify TSR using deep learning algorithms have faced challenges, such as imprecise classification models and lack of validation for prognostic significance.^19^^,^^20^ Furthermore, they encountered challenges in translating the TSR scoring protocol into an automated deep learning algorithm. Recently, a fully automated AI-based TSR quantification tool for primary colorectal cancer was developed that addresses these challenges. It enables fully automatic, quick, systematic, and quantitative analysis of TSR in whole-slide images (WSIs) without pathologists’ intervention.^21^ This tool addresses the challenge of regional heterogeneity by standardizing the analysis process and identifying the optimal analytical region according to protocol, while establishing a foundation for determining an optimal cut-off value for automated TSR assessment.^16^ Moreover, it has validated TSR as an independent prognosticator for progression-free survival and overall survival (OS), supporting the findings involving human analysts.^6^^,^^7^^,^^9^^,^^12^ In the current study, we undertake a large-scale validation of this algorithm in the UNITED cohort, a large and well-characterized, representative, multi-institutional cohort of patients with resected colon cancer.^12^ We hypothesized that the AI-based TSR quantification tool allows clinically valuable prognostic stratification of colon cancer patients independent of the disease stage and ACT.

## Materials and methods

### Digital pathology tool for TSR analysis

The fully automated TSR quantification tool has been developed and validated earlier.^22^ This tool consists of four main algorithms: tissue detection and artifact detection with quality control algorithms (open-source GrandQC tool with both deep learning-based pixel-wise segmentation algorithms), multi-class tissue segmentation algorithm for colorectal diagnostic domain (deep learning-based pixel-wise segmentation algorithm), and TSR quantification algorithm that works with output segmentation masks of different tissue classes.^21^, ^22^, ^23^ The core of the pipeline is multi-class tissue segmentation algorithm (EfficientNetB0 encoder and UNet ++ decoder). It processes WSIs in tiles of size 512 × 512 pixels at a resolution of ∼×10 objective magnification (micron-per-pixel resolution = 1.0). It allows for a pixel-wise segmentation of 11 different tissue classes (tumor epithelium, tumor stroma, necrosis, mucin, smooth muscle, mucosa, submucosa, adventitial tissue including blood vessels, dense lymphocytic infiltrates, bleeding areas) and background pixels, enabling fully automatized processing of the WSIs from primary tumors. The algorithm provides visualization of all analyzed fields overlaid on the original image for easy review by pathologists. For further analysis steps, all artificially changed areas (artifact segmentation mask) are excluded from the multi-class tissue segmentation mask, allowing robust selection of regions for downstream analysis. Subsequently, TSR quantification algorithm is applied to the modified multi-class tissue segmentation mask.

**Figure 1:**
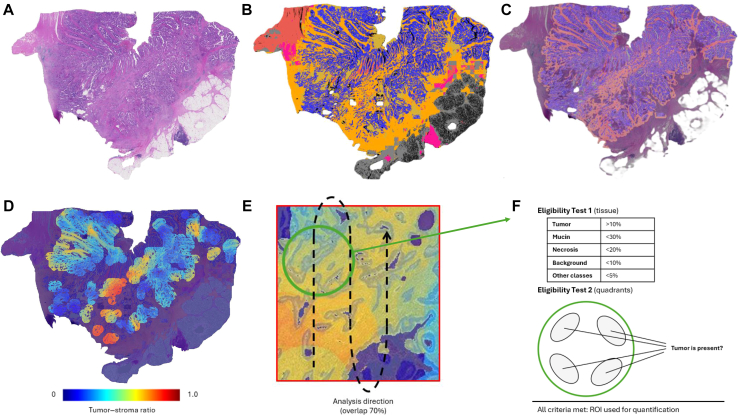
**Working principle of tumor–stroma ratio (TSR) detection algorithm.** (A) H&E-stained section of the primary colorectal cancer. (B) Precise segmentation map is generated by the backbone pixel-wise segmentation algorithm. (C) Overlay of segmentation mask on the original image generated by the algorithm, which can be easily estimated by pathologists for correctness. (D) Visual output of the TSR algorithm. (E) The systematic nature of analysis is realized in the form of a sliding window with an overlap of 70% to account for local constellations of the tissue and not to skip the eligible regions. (F) Two different eligibility tests are applied to each region: (i) for region content concerning different tissue classes and (ii) for the presence of tumor tissue in all four quadrates. Eligible regions are used for visual map construction, and all metrics are captured for calculation of the final maximal value for the slide which is used for the prediction of prognosis and further analysis. H&E, hematoxylin–eosin; ROI, region of interest.

The whole tumor region was systematically processed in regions of interest (ROIs) and TSR values were recorded to identify the highest value in those ROIs (i.e. region with highest stroma content). All regions underwent eligibility control for tumor content (four-quadrant test) and content of other tumor-associated classes (mucin, necrosis). Regions failing this eligibility test were not considered for quantification as they are thus not suited for TSR scoring. Systematic analysis of the tumor region was carried out in the sliding window mode with overlaps ensuring that all possible regions are analyzed, fully eliminating the necessity for human intervention. For more details on development, implementation, and validation, we refer to previously published studies.^21^^,^^22^ Figure 1 shows the working principle of the TSR quantification algorithm. As the TSR quantification step does not involve deep learning, and the algorithm works with pre-generated maps of WSIs, it is computationally time-effective and analyzes single WSIs within 10-30 s depending on the tumor region size. The times needed for the segmentation preprocessing step are evaluated in detail elsewhere. The AI tool analyzes WSIs in 2-5 min.^22^

### TSR quantification

TSR quantification is conducted using the formula TSR = area stroma/(area stroma + area tumor), accounting for all other tumor-associated tissue types. Thresholds for allowed content were set at <10% background, <20% necrotic debris, and <30% mucin, as established in the previous validation study.^21^ A higher TSR value, expressed as percentage, reflects a greater stroma component. According to the established visual assessment protocol, TSR scoring is carried out on the histological slide representing the T-category determination.^16^ Therefore, the selection by the pathologists remains an important factor herein. The predefined cut-off value of the standard visually determined TSR (by pathologists) is 50%, resulting in categorization of stroma-low (≤50%) and stroma-high (>50%) groups.^6^^,^^16^^,^^24^

Quantification of the automated TSR was carried out according to the same principle, steps, and parameters of Carvalho et al.^21^ The automated TSR quantification employs a segmentation algorithm capable of pixel-level, non-binary precision. Therefore, the optimal cut-off value for automated TSR was determined based on its discriminative power for OS and disease-free survival (DFS), following the approach by Mesker et al.^6^ This analysis yielded an optimal cut-off value of 80% for automated TSR (Supplementary Table S1, available at https://doi.org/10.1016/j.esmoop.2025.105934). Thereafter, receiver operating characteristic curves were constructed for the automated measured stroma percentages for both DFS and OS in a random sample of the UNITED cohort. The Youden index was calculated (Supplementary Figure S1, available at https://doi.org/10.1016/j.esmoop.2025.105934). This analysis resulted in an optimal cut-off value of 77%, which was consistent with the originally validated cut-off and therefore used as cut-off value of TSR in subsequent analysis.^21^

### Size of analysis ROI

The ROI size for visual assessment by pathologists is based on a ×10 objective viewing field of a conventional microscope, roughly corresponding to a diameter of 2 mm.^16^ However, Carvalho et al. demonstrated that a ROI of 1 mm in diameter provided superior prognostic performance in automated analysis with pixel-wise segmentation precision.^21^ Therefore, a 1-mm ROI was adopted for automated TSR quantification to maximize prognostic accuracy.

### Study cohort

This study was carried out using the UNITED cohort, including neoadjuvant therapy-naive, pathological stage II and III colon cancer patients who have undergone a complete curative resection of their primary tumor.^12^ The TSR of these patients was previously visually assessed and quantified by certified pathologists, who were specifically trained for this study through the TSR E-learning, using hematoxylin–eosin (H&E)-stained slides that were also used for determining the T-category of the tumor.^18^ The same slides were used for the automated TSR quantification. The analysis was carried out on the subset of the UNITED cohort with available digitized WSIs of the primary tumors.

### Digitization

Digitization of all pathology slides was carried out by a Panoramic 250 scanner [3DHistech, Budapest, Hungary; resolution micron per pixel (MPP) 0.39, corresponding to ∼×20 objective magnification], UFS scanner (Philips, Eindhoven, The Netherlands, ×40 magnification, MPP 0.25), or NanoZoomer (Hamamatsu Photonics, Hamamatsu City, Japan, ×20 magnification, MPP 0.45).

### Statistics

DFS was defined as the time between the date of surgery and the date of first event, i.e. recurrence (locoregional recurrence or distant metastasis) or death (from any cause). In case of no event, DFS was calculated from the date of surgery until censoring. OS was defined as the time from the date of surgery until the date of death (from any cause) or until censoring. Censoring took place when patients were free of disease and/or alive at their last follow-up appointment. All statistical analysis were carried out using IBM SPSS Statistics for Windows, version 29.0 (IBM Corp., Armonk, NY). Descriptive statistics were used for the comparison of clinicopathological variables. Chi-square tests were used to explore associations between automated TSR and pathological variables. Through reversed Kaplan–Meier analysis, the median follow-up time was calculated. Survival analyses were carried out using the Kaplan–Meier analysis with the log-rank test. Hazard ratios (HRs) with associated 95% confidence intervals (CIs) were calculated with the univariate and multivariate Cox proportional hazards model to evaluate the prognostic role of automated TSR. A two-sided *P* value of 0.05 was deemed significant.

### Ethical considerations

The original UNITED study protocol was approved by the Medical Research Ethics Committee of the Leiden University Medical Center. The UNITED study was conducted according to the Declaration of Helsinki (2013).^12^

## Results

### Baseline characteristics

The baseline clinicopathological characteristics of the analyzed cohort (*N* = 853) are shown in Table 1. Of these, 489 patients (42.7%) were of male sex and 261 (30.6%) were aged ≥75 years. A total of 428 patients (50.2%) had stage II colon cancer. The algorithm quantified the tumor as stroma-high (>77%) in 298 patients (34.9%). Two representative examples of stroma-high and stroma-low tumors analyzed by certified pathologists and the automated TSR assessment algorithm are shown in Supplementary Figure S2, available at https://doi.org/10.1016/j.esmoop.2025.105934. Distribution of demographics, surgery type, tumor morphology, and differentiation grade were equally distributed among the stroma-low (*n* = 555) and stroma-high (*n* = 298) groups. The distribution between the groups categorized by the automated TSR is shown in Table 2. Median follow-up time was 36 months (95% CI 35.4-36.5 months) and comparable between both groups (*P* = 0.006). A total of 186 events occurred, of which 87 were in the stroma-high group (29.2% of stroma-high patients; *P* < 0.001). Mostly, this concerned distant metastasis (58 stroma-high patients, 66.7% of the events).

**Table 1:** Baseline characteristics of the eligible UNITED cohort

Baseline characteristics	Total eligible UNITED cohort (*N* = 853)
Sex	
Female	364 (57.3)
Male	489 (42.7)
Age at surgery (years)	
Median age	67 (60-75)
≥75 years of age	261 (30.6)
Biopsy	
Yes, preoperative endoscopy	772 (90.5)
Yes, other method^a^	4 (0.5)
No^b^	74 (8.7)
Surgery	
Surgery year	2019 (2015-2021)
Pathological TNM stage	
Stage II	428 (50.2)
Stage III	425 (49.8)
Lymph nodes	
Examined	20 (15-28)
Positive (in pTNM stage III)	2 (2-4)
Tumor–stroma ratio (by human analysts)	
Stroma-low (≤50%)	570 (66.8)
Stroma-high (>50%)	283 (33.2)
Tumor–stroma ratio (by algorithm)	
Stroma-low (≤77%)	555 (65.1)
Stroma-high (>77%)	298 (34.9)
Follow-up	
Locoregional recurrence	27 (3.2)
Distant metastasis	131 (15.4)
New primary tumor	35 (4.1)
New primary colorectal tumor	4 (11.4)^c^
Death at end of follow-up	
Yes	103 (12.1)
Death due to colon cancer	66 (64.1)^d^
No	750 (87.9)

All variables are given as absolute numbers with associated percentages or medians with interquartile ranges.

TNM, tumor–node–metastasis.

a For example, during surgery.

b Reasons why biopsy was not taken, is almost always in the emergency setting (obstructive ileus).

c Percentage of total number of new primary tumors.

d Percentage of total number of deaths.

**Table 2:** Distribution of clinicopathological features between TSR

Variables	Stroma-low (*n* = 555)	Stroma-high (*n* = 298)	*P* value
Sex			0.453^a^
Female	242 (43.6)	122 (40.9)	
Male	313 (56.4)	176 (59.1)	
Age at surgery			0.724^a^
<75 years	219 (75.0)	88 (73.3)	
≥75 years	73 (25.0)	32 (26.7)	
Biopsy			0.828^a^
Yes	506 (91.2)	273 (91.6)	
No^b^	49 (8.8)	25 (8.4)	
Surgery type			0.348^c^
Hemicolectomy right	255 (45.9)	133 (44.6)	
Hemicolectomy left	72 (13.0)	35 (11.7)	
Sigmoidectomy^d^	169 (30.5)	97 (32.6)	
Other	59 (10.6)	33 (11.1)	
Location of tumor			0.643^a^
Right-sided tumor^e^	272 (49.0)	151 (50.7)	
Left-sided tumor	283 (51.0)	147 (49.3)	
Lymph nodes			**0.029**^a^
<12 examined	63 (11.4)	278 (93.3)	
≥12 examined	492 (88.6)	20 (6.7)	
Pathological tumor (pT) stage^f^			**<0.001**^a^
pT1-3	476 (85.8)	221 (74.2)	
pT1	9 (1.6)	0	
pT2	38 (6.8)	4 (1.3)	
pT3	429 (77.3)	217 (73.2)	
pT4	79 (14.2)	76 (25.5)	
Pathological nodal (pN) stage^f^			**0.005**^a^
pN0	291 (52.4)	137 (46.0)	
pN1	186 (33.5)	93 (31.2)	
pN2	78 (14.1)	68 (22.8)	
Tumor morphology			0.968^a^
Adenocarcinoma	502 (90.5)	268 (89.9)	
Mucinous carcinoma	46 (8.3)	25 (8.4)	
Other	7 (1.3)	5 (1.7)	
Differentiation grade^g^			0.151^a^
Well—moderate	486 (89.7)	253 (86.3)	
Poor—undifferentiated	56 (10.3)	40 (13.7)	
Grade cannot be assessed	13 (2.3)	5 (1.7)	
Pathology risk factors^h^			**0.008**^a^
None present	321 (57.8)	144 (48.3)	
≥1 present	234 (42.2)	154 (51.7)	
Extramural venous invasion (EMVI)			**0.010**^a^
Not reported	60 (10.8)	27 (9.1)	
Reported	495 (89.2)	271 (90.9)	
Yes (EMVI +)	58 (11.7)	50 (18.5)	
No (EMVI –)	437 (88.3)	221 (81.5)	
Venous invasion			0.265^a^
Not reported	82 (14.8)	53 (17.8)	
Reported	473 (85.2)	245 (82.2)	
Yes (V1)	51 (10.8)	20 (8.2)	
No (V0)	422 (89.2)	225 (91.8)	
Lymphatic invasion			0.208^a^
Not reported	34 (6.1)	10 (3.4)	
Reported	521 (93.9)	288 (96.6)	
Yes (L1)	183 (35.1)	114 (39.6)	
No (L0)	338 (64.9)	174 (60.4)	
Perineural invasion			**<0.001**^a^
Not reported	291 (52.4)	174 (58.4)	
Reported	264 (47.6)	124 (41.6)	
Yes (PN1)	36 (13.6)	34 (27.4)	
No (PN0)	228 (86.4)	90 (72.6)	
Microsatellite instability/mismatch repair (MMR) status			0.107^a^
Not determined	190 (34.2)	105 (35.2)	
Determined	365 (65.8)	193 (64.8)	
Microsatellite stable (MSS)/MMR proficient (pMMR)	294 (80.5)	166 (86.0)	
Microsatellite instable (MSI)/MMR deficient (dMMR)	71 (19.5)	27 (14.0)	
Pathological TNM stage			0.072^a^
Stage II	291 (52.4)	137 (46.0)	
Stage III	264 (47.6)	161 (54.0)	
Adjuvant chemotherapy			0.052^a^
Yes	263 (47.4)	162 (54.4)	
No	292 (52.6)	136 (45.6)	
Adjuvant chemotherapy per pathological TNM stage			**0.025**^a^
Stage II + no adjuvant chemotherapy	243 (43.8)	101 (33.9)	
Stage II + adjuvant chemotherapy	48 (8.6)	36 (12.1)	
Stage III + no adjuvant chemotherapy	49 (8.8)	35 (11.7)	
Stage III + adjuvant chemotherapy	215 (38.7)	126 (42.3)	

All variables are given as absolute numbers with associated percentages.

*P* values in bold show statistical significance.

PN, perineural invasion; TNM, tumor–node–metastasis; TSR, tumor–stroma ratio; UICC, Union for International Cancer Control.

a Calculated using the chi-square test.

b Reasons why biopsy was not taken, is almost always in the emergency setting (obstructive ileus).

c Calculated using the chi-square test for the three most common and here presented operation types: hemicolectomy right, hemicolectomy left, and sigmoidectomy.

d Other surgery types include a (sub)total colectomy, high anterior resection, or transversectomy.

e A right-sided tumor is defined as a colon carcinoma in the cecum, colon ascendens, flexura hepatica, or colon transversum.

f All variables are converted to the UICC TNM version 5 (1997).

g Differentiation grade is variously registered as separate or combined subgroups; this is then categorized into combined grades, i.e. well—moderate or poor—undifferentiated.

h Pathological risk factors are stated in Table 2. Presence of a risk factor is defined as at least one of the registered risk factors. Absence is the absence of registered risk factors, as not all risk factors are registered.

### Disease-free survival

**Figure 2:**
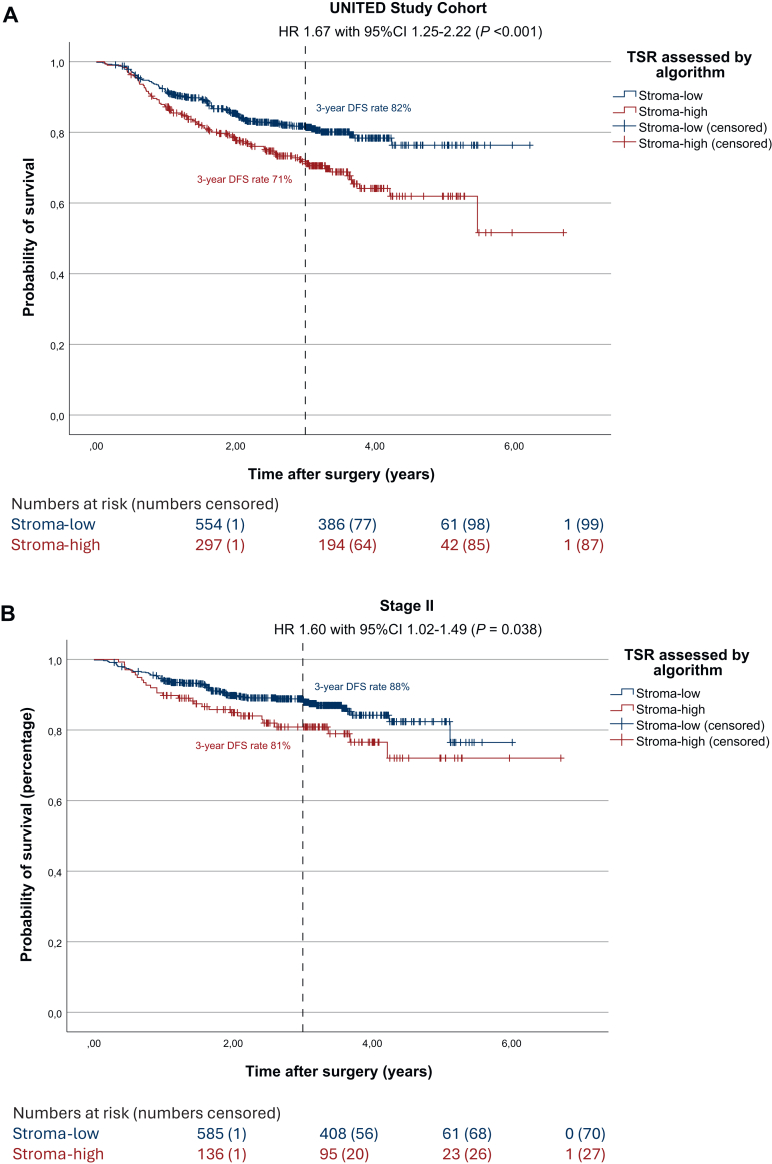

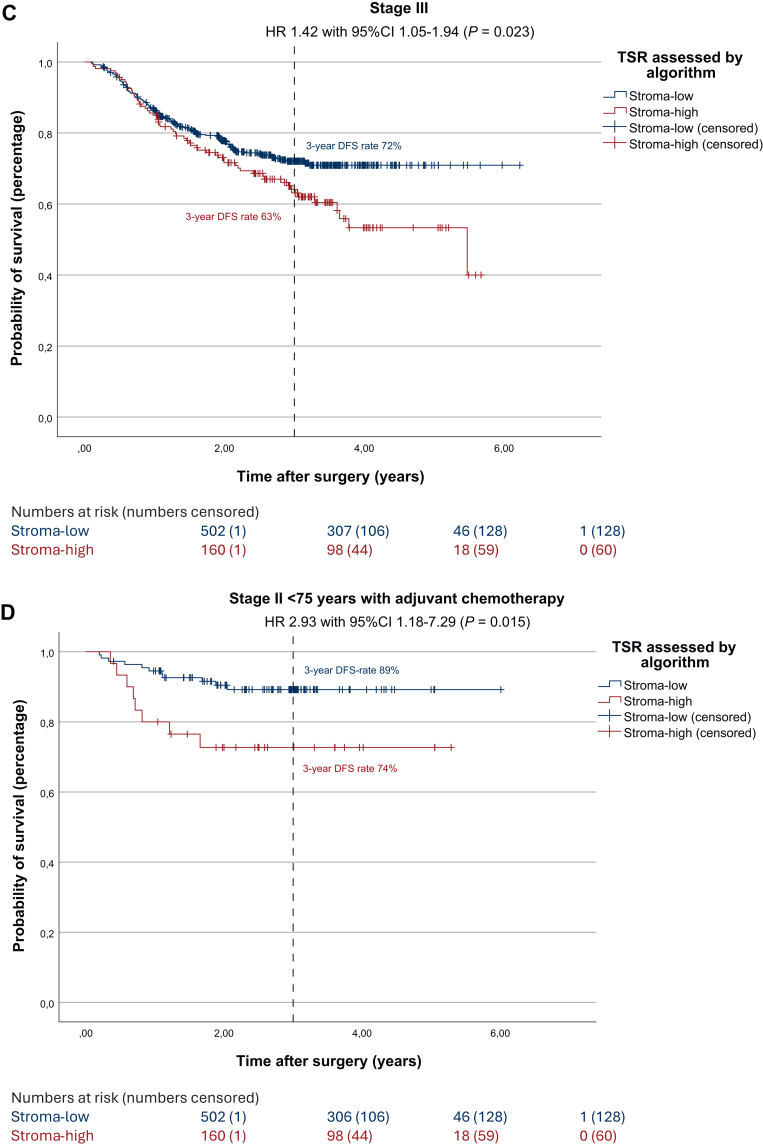

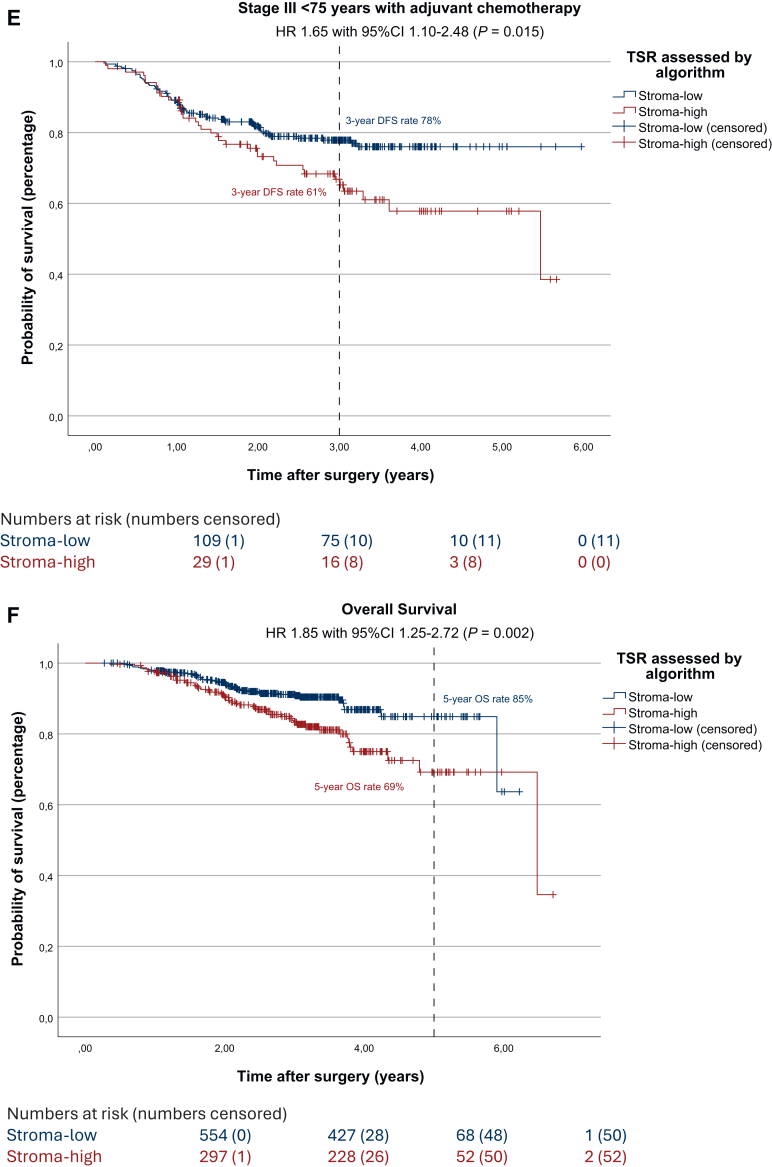
**Survival effect of automated TSR in the UNITED cohort and subgroup analyses.** (A) Kaplan–Meier analysis and log-rank test showing worse 3-year DFS rates for stroma-high patients (3-year DFS 71% and 82%, respectively; HR 1.67, 95% CI 1.25-2.22, *P* < 0.001). (B) Kaplan–Meier analysis and log-rank test in stage II patients, showing worse 3-year DFS rates for stroma-high patients (3-year DFS 81% and 88%, respectively; HR 1.60, 95% CI 1.02-2.49, *P* = 0.038). (C) Kaplan–Meier analysis and log-rank test in stage III patients, showing worse 3-year DFS rates for stroma-high patients (3-year DFS 63% and 72%, respectively; HR 1.42, 95% CI 1.05-1.94, *P* = 0.023). (D) Kaplan–Meier analysis and log-rank test in stage II patients receiving adjuvant chemotherapy, showing worse 3-year DFS rates for stroma-high patients despite treatment, after correction for age (3-year DFS 74% and 89%, respectively; HR 2.93, 95% CI 1.18-7.29, *P* = 0.015). (E) Kaplan–Meier analysis and log-rank test in stage III patients receiving adjuvant chemotherapy, showing worse 3-year DFS rates for stroma-high patients despite treatment, after correction for age (3-year DFS 61% and 78%, respectively; HR 1.65, 95% CI 1.10-2.48, *P* = 0.015). (F) Kaplan–Meier analysis and log-rank test showing worse 5-year overall survival rates for stroma-high patients (5-year OS 69% and 85%, respectively; HR 1.85, 95% CI 1.25-2.27, *P* = 0.002). CI, confidence interval; DFS, disease-free survival; HR, hazard ratio; OS, overall survival; TSR, tumor–stroma ratio.

The results of the survival analysis examining the visually assessed TSR and its impact of DFS in this subset were identical to those observed in the full UNITED cohort (shown in Supplementary Figure S3, available at https://doi.org/10.1016/j.esmoop.2025.105934). The univariate survival analysis regarding automated TSR resulted in a statistically significant worse DFS in stroma-high patients compared with stroma-low patients (3-year DFS rate 71% and 82%, respectively; HR 1.67, 95% CI 1.25-2.22, *P* < 0.001; Figure 2A). In multivariate analysis DFS remained worse for automatically assessed stroma-high patients, confirming the independent prognostic effect of the automated TSR on DFS (HR 1.46, 95% CI 1.07-1.95, *P* = 0.016; Supplementary Table S2, available at https://doi.org/10.1016/j.esmoop.2025.105934). Worse DFS in automatically assessed stroma-high patients remained significant in stage II (3-year DFS rate 81% and 88%, respectively; HR 1.60, 95% CI 1.02-2.49, *P* = 0.002; Figure 2B) and stage III colon cancer patients (3-year DFS rate 63% and 72%, respectively; HR 1.42, 95% CI 1.05-1.94, *P* = 0.023; Figure 2C).

Furthermore, we analyzed potential added benefit from ACT in stage II and III patients and influence of the automated TSR herein on DFS. Within the UNITED cohort, in stage II patients who did receive ACT (*n* = 140), a significantly worse DFS was seen after correction for age (3-year DFS rate 74% and 89%, respectively; HR 2.93, 95% CI 1.18-7.29, *P* = 0.015; Figure 2D). In the stage III group receiving ACT (*n* = 418), DFS rates were significantly worse for automatically assessed stroma-high patients as well after correction for age (3-year DFS rate 65% and 79%, respectively; HR 1.76, 95% CI 1.22-2.54, *P* = 0.002; Figure 2E).

### Overall survival

A total of 103 deaths were recorded, of which 53 (51.5%) were in the automatically assessed stroma-high group (17.8% of stroma-high patients; *P* < 0.001). Stroma-high patients showed a significantly worse OS as well, compared with stroma-low patients (5-year OS rate 69% and 85%, respectively; HR 1.85, 95% CI 1.25-2.72, *P* = 0.002; Figure 2F). In this subset of the UNITED cohort, visually assessed stroma-high tumors were associated with significantly worse OS compared with stroma-low tumors (5-year OS rate 69% and 83%, respectively; HR 1.60, 95% CI 1.08-2.38, *P* = 0.017; Supplementary Figure S3, available at https://doi.org/10.1016/j.esmoop.2025.105934). However, this was not observed in the full UNITED cohort.^12^ When compared with automated TSR, shown in Supplementary Figure S4, available at https://doi.org/10.1016/j.esmoop.2025.105934, the automated method demonstrated a slightly greater discriminatory power than the visual assessment (HR 1.85, 95% CI 1.25-2.27, *P* = 0.002 for automated TSR versus HR 1.60, 95% CI 1.08-2.38, *P* < 0.017 for visually assessed TSR).

### Region of interest (ROI) post hoc exploratory analysis

The analysis was repeated using different ROI sizes of 1.5 mm and 2.0 mm. The 1.5-mm ROI size revealed a significantly worse DFS for stroma-high patients compared with stroma-low patients in univariate analysis (3-year DFS rate 72% and 81%, respectively; HR 1.64, 95% CI 1.23-2.18, *P* < 0.001; Supplementary Figure S5A, available at https://doi.org/10.1016/j.esmoop.2025.105934). However, this association was not seen after adjustment for significant variables in the multivariate analysis (HR 1.33, 95% CI 0.98-1.81, *P* = 0.065). Using the 2.0-mm ROI, univariate analysis also revealed significantly worse DFS for stroma-high patients (3-year DFS rate 71% and 82%, respectively; HR 1.78, 95% CI 1.29-2.45, *P* < 0.001; Supplementary Figure S6A, available at https://doi.org/10.1016/j.esmoop.2025.105934), which did not persist in the multivariate analysis (HR 1.24, 95% CI 0.89-1.74, *P* = 0.221). These data are shown in Supplementary Figures S5 and S6, available at https://doi.org/10.1016/j.esmoop.2025.105934.

Supplementary Figures S7 and S8, available at https://doi.org/10.1016/j.esmoop.2025.105934, illustrate how ROI size influences the visual segmentation and stromal percentage. A ROI size of 1.0 mm in diameter provides the most optimal assessment for automated TSR analysis, in line with the original publication.^21^

## Discussion

This study validated a fully automated and accurate algorithm for the quantification of the TSR in colon cancer. While TSR has been established as an independent prognosticator in colon cancer, most published studies have relied on visual assessment by human observers.^12^^,^^14^^,^^25^ Certain levels of interobserver variability are natural to assessment by pathologists.^16^ Therefore, automatic algorithms as assistive devices for pathologists are a field of active exploration as a source of analysis objectivization.^26^, ^27^, ^28^ The digital pathology tool presented here introduces standardization in automated TSR quantification and validated the optimal ROI size for automated scoring, supporting the conclusions previously drawn by Smit et al.^21^^,^^29^ Importantly, no human interference is required at the analytical stage, and only validation of the outputs, reducing effort when compared with single case analyses. In the study by Carvalho et al., a ROI of 1 mm proved to be the optimal analytical window size extracting the most prognostic value from TSR measurements and making more tumor regions eligible for analysis.^21^ This pattern was also observed in our current study, showing that the prognostic value of TSR remains significant in multivariate analysis when using a 1-mm ROI and not when using ROI sizes of 1.5 mm or 2 mm, despite significant associations in the univariate analysis.

In this study, we applied automatic TSR assessment to one of the largest and well-characterized colon cancer cohorts available (multi-institutional international UNITED study). Our results validate that automated TSR scoring reflects the aggressive behavior of stroma-high tumors, in line with findings from studies based on visual TSR assessment.^9^^,^^12^^,^^14^ Patients with stroma-high tumors show worse 3-year DFS rates compared with stroma-low tumors, independent of other established prognostic high-risk factors. Moreover, our results suggest that TSR has a potential predictive value in patients scheduled for ACT, a finding also supported by the UNITED study.^12^ Here, correction for age was carried out due to a significant difference in mean age between patients who received ACT and those who did not (64 years and 71 years, respectively, with *P* < 0.001 in stage II; 65 years and 77 years, respectively, with *P* < 0.001 in stage III). As a follow-up, the UNITED II study has been initiated to validate TSR’s possible predictive value for treatment response with sufficient statistical power. Although the original UNITED study did not find a statistically significant difference in OS between visually assessed stroma-high and stroma-low tumors, it did observe a trend toward worse OS for stroma-high patients. Our study did demonstrate a significant difference in OS using both automated and visual TSR assessments, with automated TSR quantification showing a slightly greater discriminatory power. The current study therefore highlights the comparable prognostic utility of automated TSR scoring, with a potentially enhanced ability to differentiate OS outcomes. However, we demonstrate clear and significant prognostic values for more relevant cancer-specific endpoints, such as DFS.

Besides existing challenges, previous efforts to quantify TSR using deep learning algorithms have shown promising results. A study by Smit et al. showed good correlations between visual TSR estimation and semi-automated TSR scores, despite some discrepancies. However, due to the semi-automated nature of the TSR assessment, annotations were needed in advance by one of the observers to highlight the ROI and facilitate consensus on stromal percentage estimation.^29^ Similarly, the deep learning-based approach for rectal cancer proposed by Geessink et al. required the position of a user-provided stroma hotspot as input to assess TSR, highlighting the need for initial user input in both methods.^17^ Previous studies have shown that human observers tend to score systematically higher than computer algorithms when quantifying features such as the percentage of malignant nuclei.^30^^,^^31^ In contrast, automated assessment of the TSR generally yields higher stroma percentages compared with visual evaluation by pathologists. Consequently, applying the conventional 50% cut-off to automated TSR scores potentially results in poorer agreement with visual TSR assessments, as indicated by lower kappa values.^17^ These findings highlight that measurements provided by pathologists might not be directly comparable to mathematically precise measurements of pixel-level AI algorithms, a consistent observation from earlier studies.^17^^,^^21^^,^^30^^,^^31^

Therefore, certain methodological differences between visual and automated TSR scoring should be acknowledged. Visual TSR assessment is typically carried out on the most invasive part of the tumor, which is also used by pathologists to determine the T-category. This approach allows for consistent exclusion of non-relevant tissue classes and overcomes the subjectivity inherent to manual assessment. Automated TSR quantification employs a segmentation algorithm capable of pixel-level, non-binary precision. This precision of the algorithm is, however, balanced by a limitation and source of analytical instability: sensitivity to variations in staining quality and the presence of artifacts. While experienced pathologists can adjust their evaluation to such inconsistencies, the performance of AI-based algorithms may be adversely affected. This limitation was also evident when applying the algorithm to scanned slides from the UNITED study, where sensitivity to artifacts and suboptimal staining quality impacted the algorithm’s ability to assess areas of certain slides. We applied a rigorous quality control using state-of-the-art tools for digital pathology.^22^^,^^23^ With this, areas with artifacts are being masked from analysis to prevent false results. However, this approach also excludes potentially relevant areas from analysis, while this is mostly not the case in course of visual assessment. Pathologists have high levels of tolerance to artificial changes and can analyze the tissue even in the context of severe artificial changes.

Furthermore, the algorithm is capable of analyzing the entire tumor across multiple WSIs. In the UNITED cohort, however, only a single slide per patient was available. Whole tumor analysis requires scanning and processing all WSIs, which demands additional time and effort. Still, the algorithm analyzes single WSIs within 10-30 s at the TSR analysis step on segmentation masks, without the need for manual annotation, resulting in a full WSI analysis of 2-5 min per patient.^22^ This shows that automated TSR assessment is more rapid than visual TSR assessment, which typically takes trained pathologists 2-3 min per slide. This highlights the significant advantage of automated TSR scoring in terms of speed and efficiency. However, scoring the TSR across the whole tumor area may yield lower TSR scores compared with a single hotspot, complicating direct comparisons.^32^ Importantly, this study is the first to compare an automated approach following the protocol by van Pelt et al. and to validate both its ROI selection strategy and proposed cut-off value.^16^

Our study is not without limitations. As a retrospective analysis, prospective validation is necessary to confirm our findings. Additionally, our assessment of ACT effects may be subject to bias, as the UNITED study was not originally designed or powered to evaluate this specifically. Despite stratification for known risk factors such as age, the strong a priori prognostic impact may still confound the results. Additionally, several scanned slides contained artifacts or regions that were out of focus, rendering large portions, or in some cases entire slides, unsuitable for analysis. As a result, of the 1072 available slides of the UNITED cohort, 219 slides could not be analyzed using the automated method but remained assessable through conventional visual TSR scoring by pathologists. Finally, regarding algorithm design, high-grade dysplasia was initially modeled as a separate class in the segmentation algorithm.^22^ For tumor detection applications, such as biopsies, high-grade dysplasia was merged with invasive carcinoma. For TSR quantification in both our previous work and current study, regions with high-grade dysplasia are excluded during slide-eligibility screening using a background-threshold filter.^21^ While this approach performed robustly during development, some regions with high-grade dysplasia may bypass the filter. Nevertheless, as such regions are inherently stroma-low, they are unlikely to materially affect the calculated TSR values.

Looking ahead, the clinical implementation of automated TSR scoring offers a practical opportunity to improve personalized cancer care. With digital slides available, the algorithm can process cases quickly and consistently, allowing for rapid, objective, and reproducible scoring, reducing the time and effort required from pathologists. Therefore, in low-income countries with often limited access to specialized pathology expertise, automated TSR assessment could provide an important opportunity to strengthen cancer diagnostics. However, this does require access to slide scanners, which are costly and therefore not widely available. The method is well suited for large-scale application, as the algorithm requires no manual annotation and can process WSIs within seconds.

In clinical practice, the pathologists would retain full oversight and determine whether a case is suitable for automated TSR scoring. In this setting, the AI tool would function as a supportive, efficiency-enhancing component within the diagnostic process, while manual review would be reserved for slides of insufficient quality or unusual histology.

With the rapid developments in digital pathology and AI, future developments may enable the simultaneous assessment of multiple histopathological biomarkers within a unified algorithmic framework. Integrating TSR with complementary features such as tumor-infiltrating lymphocytes or tumor-budding could further enhance prognostic precision by capturing distinct biological aspects, including stromal composition, immune response, and invasive potential. Although such integration was beyond the scope of the current study, this represents a promising direction for future research. The growing maturity of the AI tool capable of reliably quantifying these parameters now makes multi-parametric, tissue-based biomarker assessment technically feasible. Ongoing work therefore aims to combine automated TSR with other validated AI-derived metrics to evaluate their additive or synergistic prognostic value and support translation into standardized, clinically applicable pathology workflows.

## Conclusion

In this study, we validated a robust and clinically applicable tool for fully automated TSR quantification in primary colon cancer. We furthermore carried out a *post hoc* analysis with determination of an optimal ROI size and optimal cut-off value for automatic assessment. Importantly, we demonstrated the independent prognostic value of automated TSR for both DFS and OS, as well as the influence of TSR on the potential benefit of ACT, emphasizing its clinical relevance. This study also addresses key challenges encountered in previous attempts to automate TSR assessment, such as discrepancies between human and algorithmic scoring and variability in cut-off determination. The significant findings regarding the clinical value of TSR evaluation in the UNITED study, which were presented to the International Collaboration on Cancer Reporting, prompted the recommendation to validate an automated method for TSR assessment. As pathology workflows continue to evolve toward full digitization, this validation is crucial to enhance standardization, reproducibility, and efficiency in TSR assessment. Automation not only enables faster turnaround times in clinical practice but also supports the broader adoption of TSR as a routine prognostic biomarker, seamlessly integrating into the digital infrastructure of modern pathology laboratories.

## UNITED collaboration

F. Heilijgers, G.W. van Pelt, K. C. M. J. Peeters, M. A. Smit, M. Polack, R. A. E. M. Tollenaar, W. E. Mesker, E. Meershoek-Klein Kranenbarg, A. G. H. Roodvoets, H. Putter, A. S. L. P. Crobach, H. Gelderblom, G. R. Vink, M. Koopman, M. M. Lacle, L. Mekenkamp, F. Beverdam, J. Jansen, M. Strous, N. W. J. Bulkmans, J. van Baarlen, R. Hoekstra, M. Sie, N. E. Leeuwis-Fedorovich, K. A. Talsma, E.Witteveen, M. P. Hendriks, A. Bordbar, R. Arensman, V. Terpstra, A. van Tilburg, M. Vermaas, J. W. T. Dekker, J. H. van Krieken, E. M. V. de Cuba, M. Fontes-Sousa, A. Raimundo, P. Borralho Nunes, J. Cruz, R. Souza da Silva, M. J. Brito, M. Cuatrecasas, M. Teresa Rodrigo, I. Archilla Sanz, A. Zucchiatti, S. Landolfi, S. Simonetti, S. Kjaer-Frifeldt, J. Lindebjerg, R. Feakins, S. Natu, R. Al Dieri, E. Dequeker, G. Petrushevska, P. Zdravkovski, P. Karagjozov, D. Dzambaz, S. Antovic, M. Bogdanovska Todorovska, S. Kostadinova Kunovska, and G. Gasljevic.
