# Supplementary figures and images for: Automating tumor–stroma ratio quantification in colon cancer patients from the UNITED study
Source: ESMO Open. 2025 Dec 30;11(1):105934. doi: 10.1016/j.esmoop.2025.105934 (PMC12804037; doi:10.1016/j.esmoop.2025.105934)

A

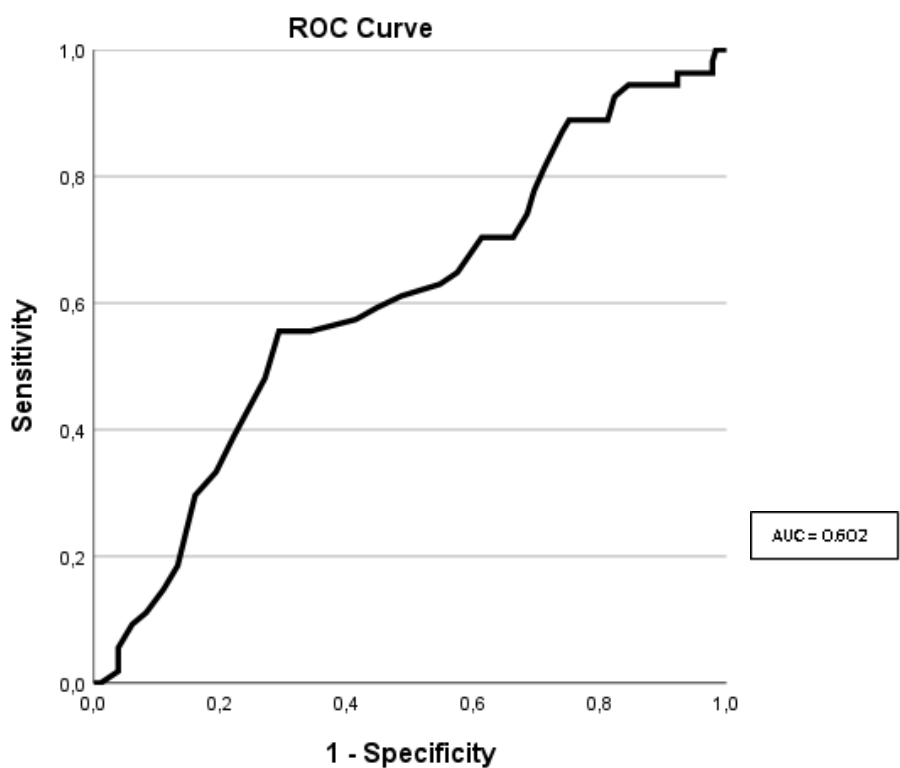

B

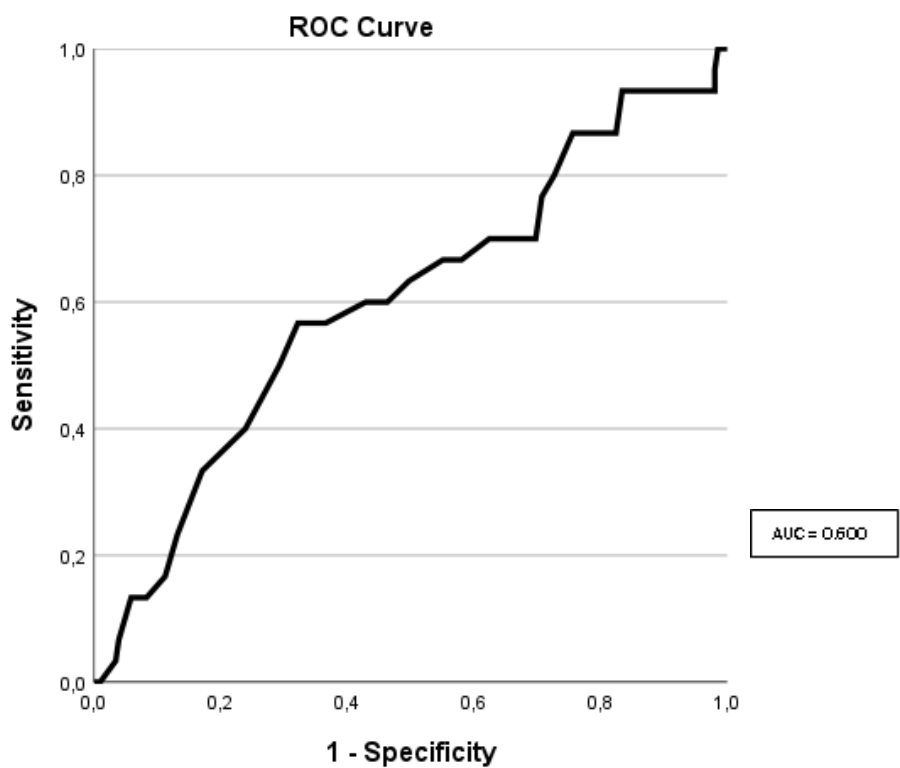

Supplement: Supplementary Figure 1 [file mmc1.pdf]

A

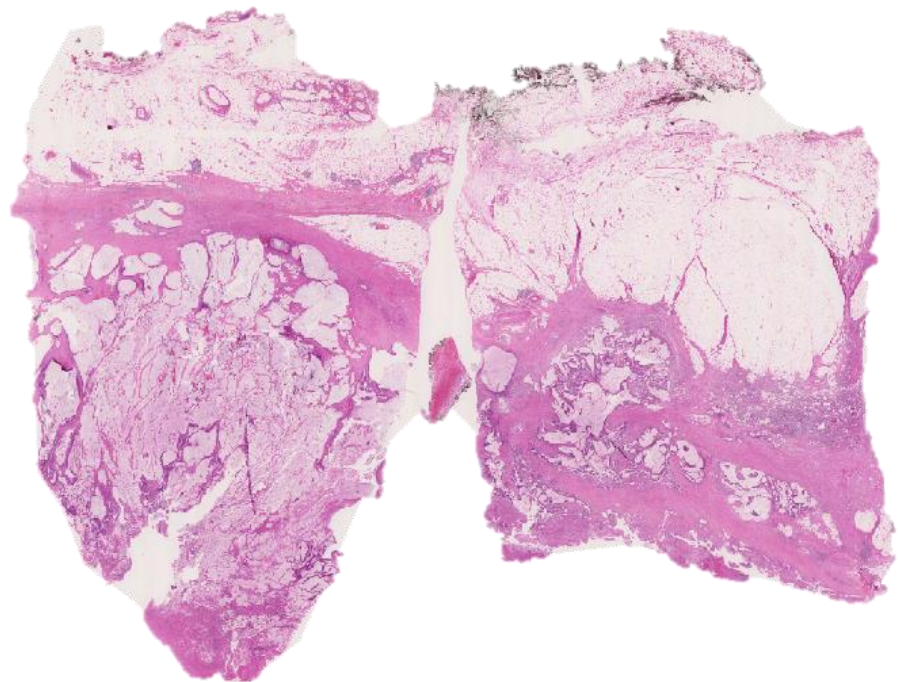

B

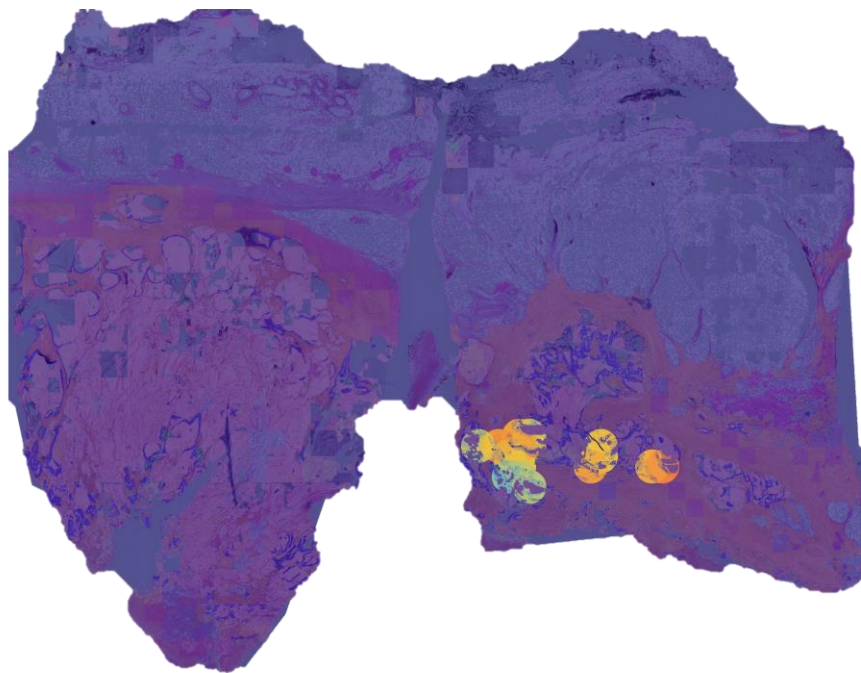

C

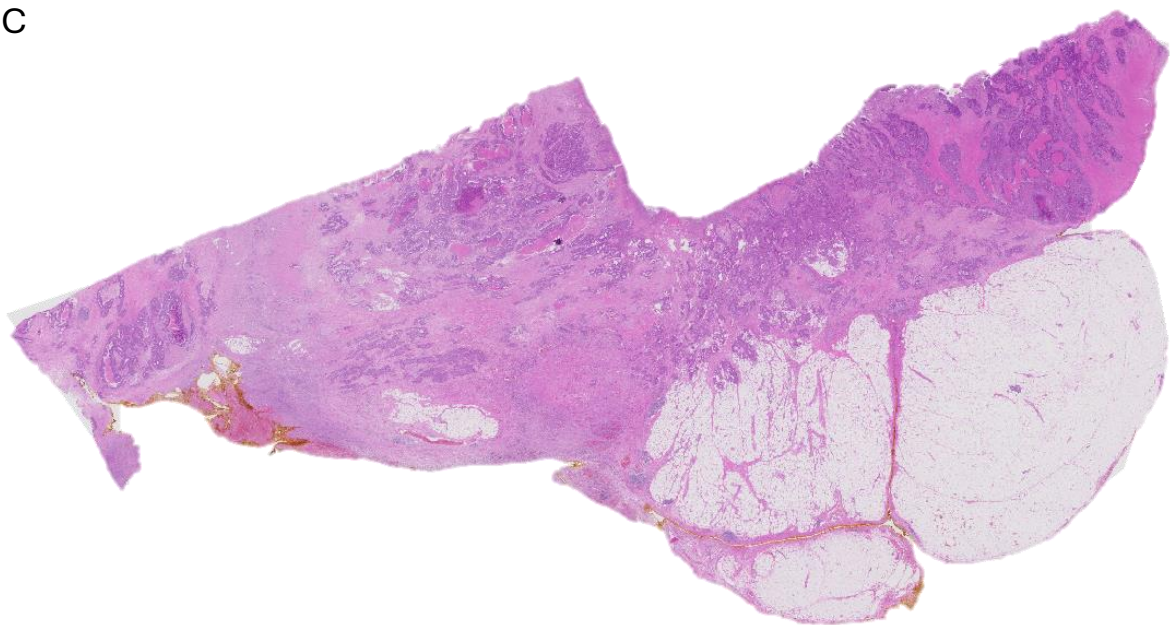

D

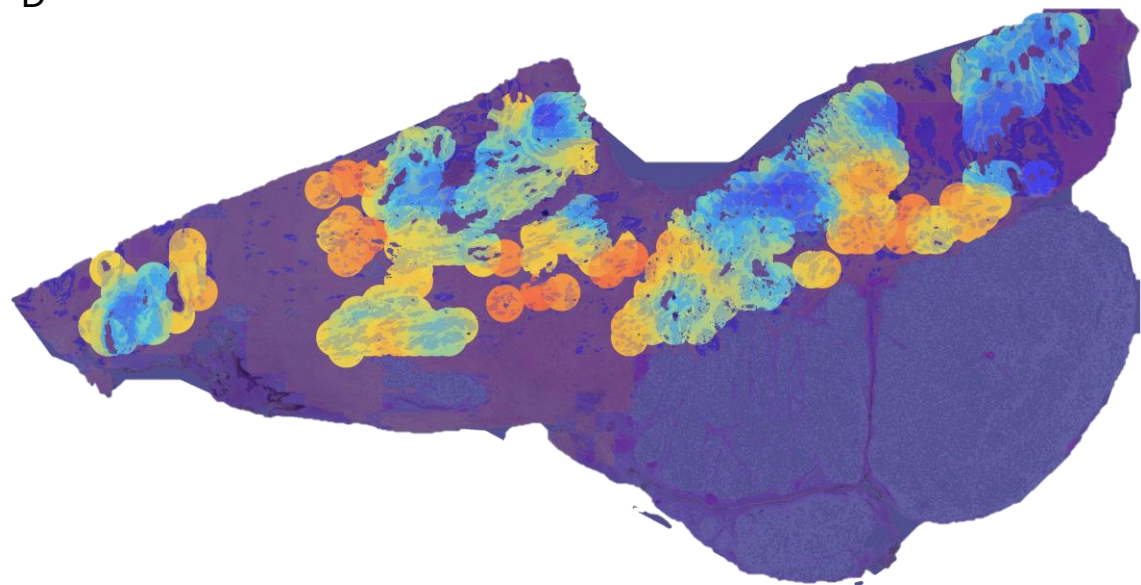

Supplement: Supplementary Figure 2 [file mmc2.pdf]

A

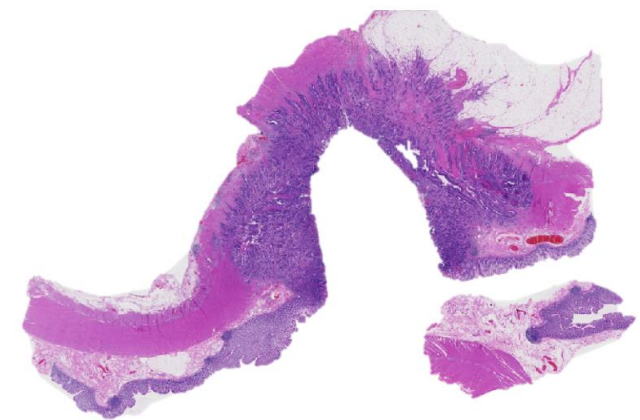

B

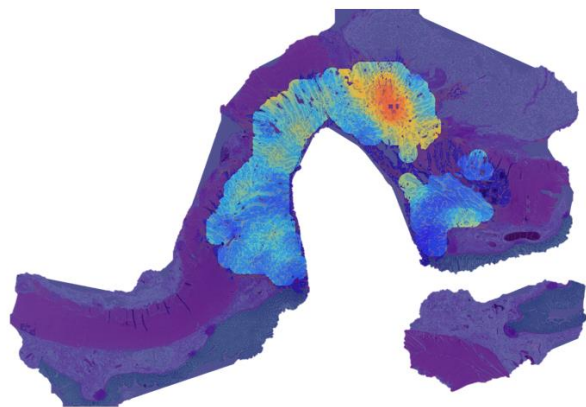

C

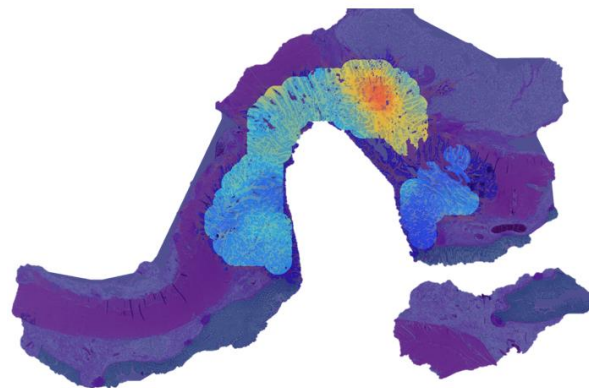

D

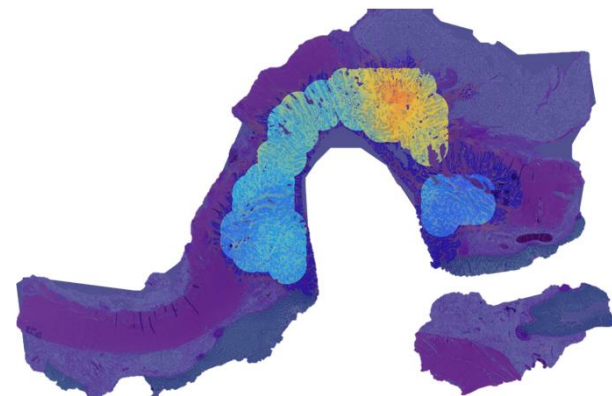

E

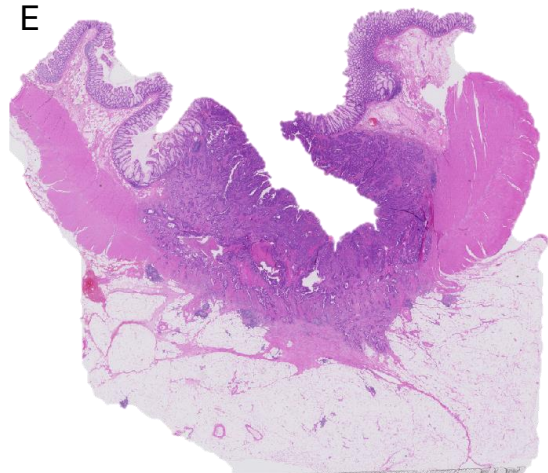

F

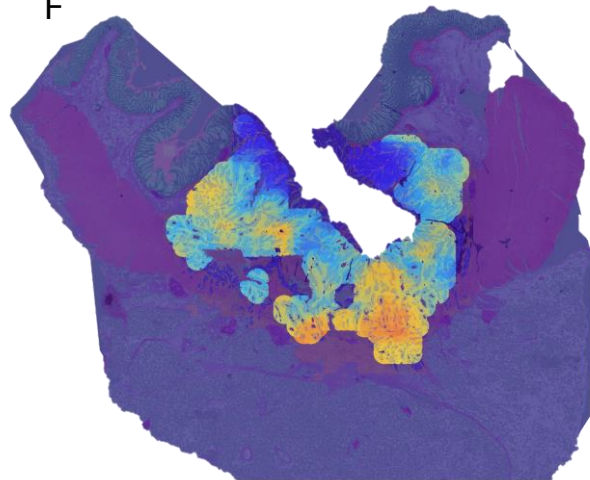

G

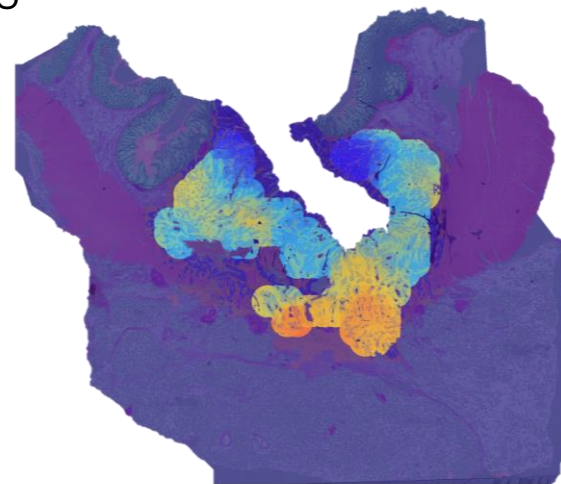

H

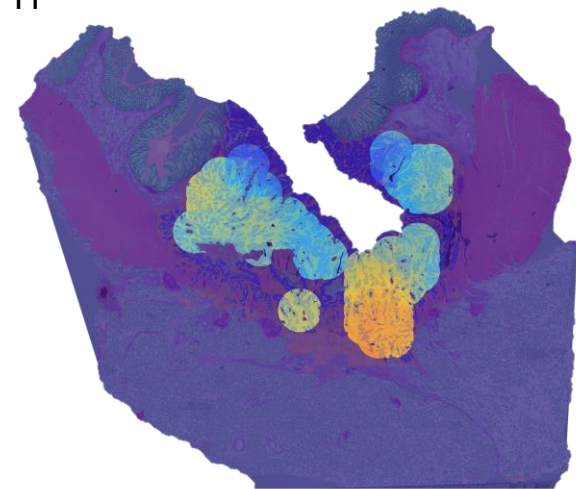

Supplement: Supplementary Figure 7 [file mmc7.pdf]
