# Supplementary material for: Automating tumor–stroma ratio quantification in colon cancer patients from the UNITED study
Source: ESMO Open. 2025 Dec 30;11(1):105934. doi: 10.1016/j.esmoop.2025.105934 (PMC12804037; doi:10.1016/j.esmoop.2025.105934)
Supplement: Supplementary Figure 3 [file mmc3.pdf]

A

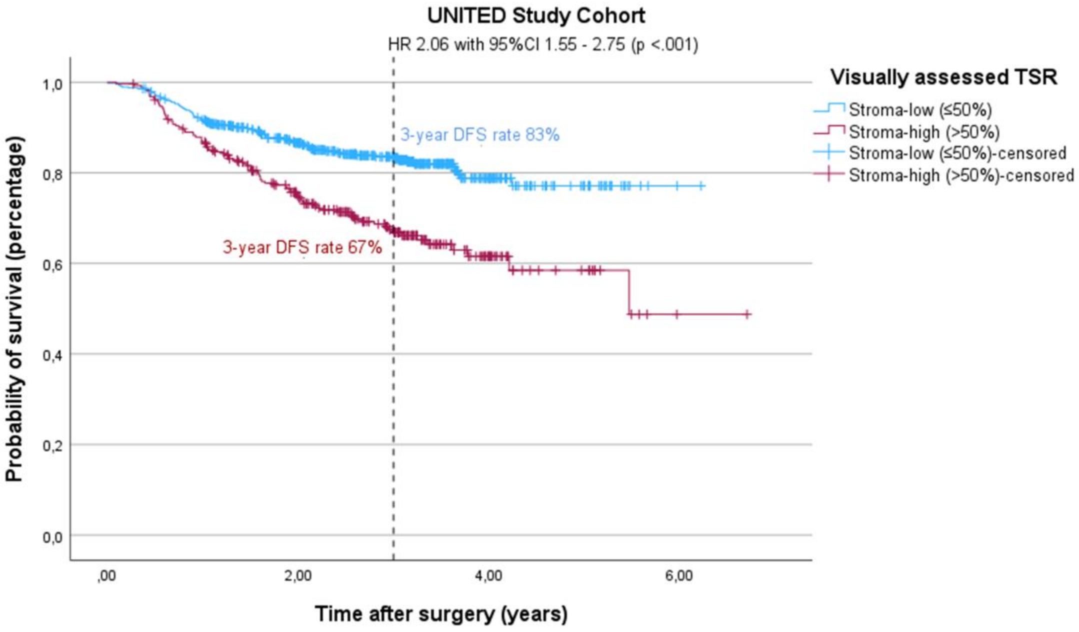

Numbers at risk (numbers censored)

Stroma-low	569 (1)	403 (73)	71 (94)	1 (95)
Stroma-high	282 (1)	178 (68)	32 (89)	1 (91)

B

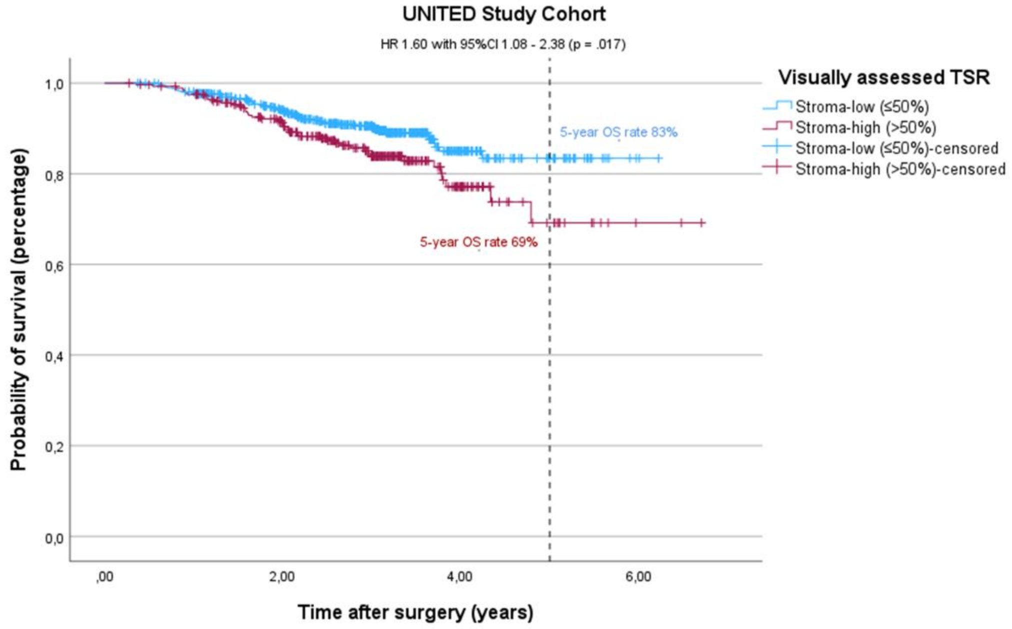

Numbers at risk (numbers censored)

Stroma-low	569 (0)	436 (31)	78 (55)	1 (57)
Stroma-high	282 (0)	218 (23)	42 (43)	2 (45)
