# Supplementary material for: Automating tumor–stroma ratio quantification in colon cancer patients from the UNITED study
Source: ESMO Open. 2025 Dec 30;11(1):105934. doi: 10.1016/j.esmoop.2025.105934 (PMC12804037; doi:10.1016/j.esmoop.2025.105934)
Supplement: Supplementary Figure 5 [file mmc5.pdf]

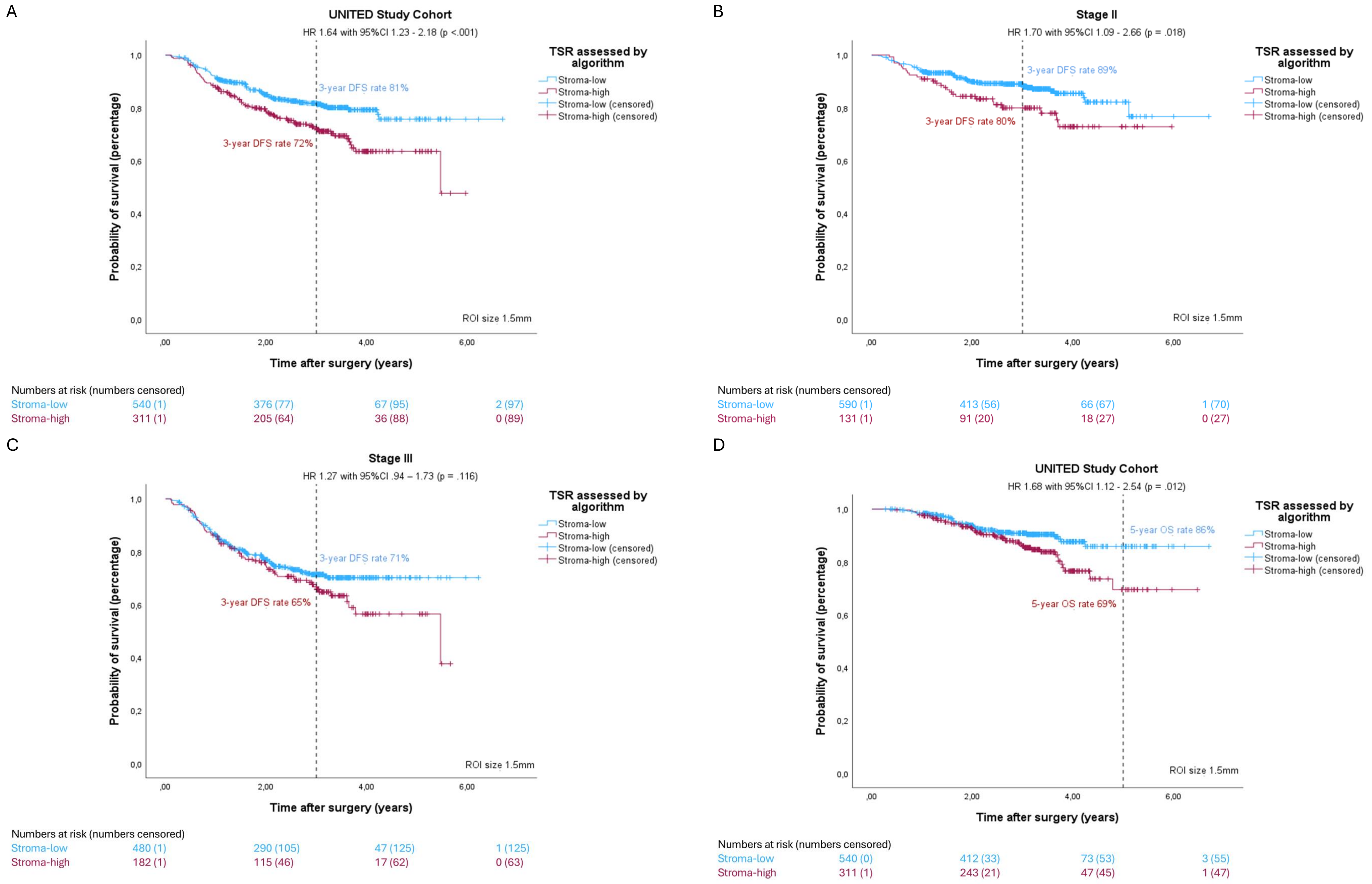
A
UNITED Study Cohort
HR 1.64 with 95%CI 1.23 - 2.18 (p <.001)
TSR assessed by algorithm
Stroma-low
Stroma-high
Stroma-low (censored)
Stroma-high (censored)
3-year DFS rate 81%
3-year DFS rate 72%
ROI size 1.5mm
Probability of survival (percentage)
Time after surgery (years)
Numbers at risk (numbers censored)
Stroma-low 540 (1) 376 (77) 67 (95) 2 (97)
Stroma-high 311 (1) 205 (64) 36 (88) 0 (89)
B
Stage II
HR 1.70 with 95%CI 1.09 - 2.66 (p = .018)
3-year DFS rate 89%
3-year DFS rate 80%
Numbers at risk (numbers censored)
Stroma-low 590 (1) 413 (56) 66 (67) 1 (70)
Stroma-high 131 (1) 91 (20) 18 (27) 0 (27)
C
Stage III
HR 1.27 with 95%CI .94 – 1.73 (p = .116)
3-year DFS rate 71%
3-year DFS rate 65%
Numbers at risk (numbers censored)
Stroma-low 480 (1) 290 (105) 47 (125) 1 (125)
Stroma-high 182 (1) 115 (46) 17 (62) 0 (63)
D
UNITED Study Cohort
HR 1.68 with 95%CI 1.12 - 2.54 (p = .012)
5-year OS rate 86%
5-year OS rate 69%
Numbers at risk (numbers censored)
Stroma-low 540 (0) 412 (33) 73 (53) 3 (55)
Stroma-high 311 (1) 243 (21) 47 (45) 1 (47)
