# Supplementary material for: Automating tumor–stroma ratio quantification in colon cancer patients from the UNITED study
Source: ESMO Open. 2025 Dec 30;11(1):105934. doi: 10.1016/j.esmoop.2025.105934 (PMC12804037; doi:10.1016/j.esmoop.2025.105934)
Supplement: Supplementary Figure 6 [file mmc6.pdf]

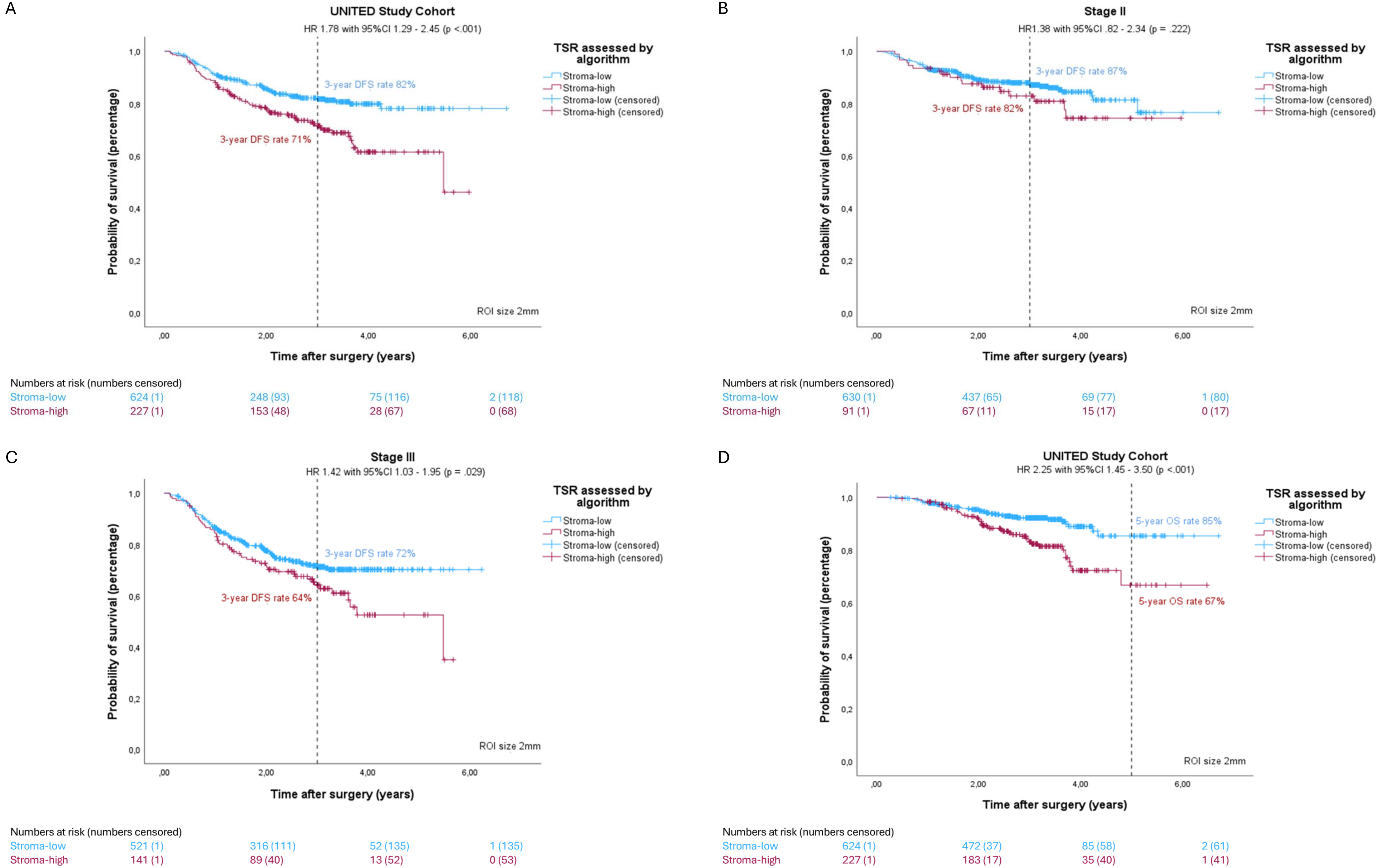
A
UNITED Study Cohort
HR 1.78 with 95%CI 1.29 - 2.45 (p <.001)
TSR assessed by algorithm
Stroma-low
Stroma-high
Stroma-low (censored)
Stroma-high (censored)
3-year DFS rate 82%
3-year DFS rate 71%
Probability of survival (percentage)
1,0
0,8
0,6
0,4
0,2
0,0
ROI size 2mm
,00
2,00
4,00
6,00
Time after surgery (years)
Numbers at risk (numbers censored)
Stroma-low 624 (1) 248 (93) 75 (116) 2 (118)
Stroma-high 227 (1) 153 (48) 28 (67) 0 (68)
B
Stage II
HR1.38 with 95%CI .82 - 2.34 (p = .222)
TSR assessed by algorithm
Stroma-low
Stroma-high
Stroma-low (censored)
Stroma-high (censored)
3-year DFS rate 87%
3-year DFS rate 82%
Probability of survival (percentage)
1,0
0,8
0,6
0,4
0,2
0,0
ROI size 2mm
,00
2,00
4,00
6,00
Time after surgery (years)
Numbers at risk (numbers censored)
Stroma-low 630 (1) 437 (65) 69 (77) 1 (80)
Stroma-high 91 (1) 67 (11) 15 (17) 0 (17)
C
Stage III
HR 1.42 with 95%CI 1.03 - 1.95 (p = .029)
TSR assessed by algorithm
Stroma-low
Stroma-high
Stroma-low (censored)
Stroma-high (censored)
3-year DFS rate 72%
3-year DFS rate 64%
Probability of survival (percentage)
1,0
0,8
0,6
0,4
0,2
0,0
ROI size 2mm
,00
2,00
4,00
6,00
Time after surgery (years)
Numbers at risk (numbers censored)
Stroma-low 521 (1) 316 (111) 52 (135) 1 (135)
Stroma-high 141 (1) 89 (40) 13 (52) 0 (53)
D
UNITED Study Cohort
HR 2.25 with 95%CI 1.45 - 3.50 (p <.001)
TSR assessed by algorithm
Stroma-low
Stroma-high
Stroma-low (censored)
Stroma-high (censored)
5-year OS rate 85%
5-year OS rate 67%
Probability of survival (percentage)
1,0
0,8
0,6
0,4
0,2
0,0
ROI size 2mm
,00
2,00
4,00
6,00
Time after surgery (years)
Numbers at risk (numbers censored)
Stroma-low 624 (1) 472 (37) 85 (58) 2 (61)
Stroma-high 227 (1) 183 (17) 35 (40) 1 (41)
