# Supplementary material for: Automating tumor–stroma ratio quantification in colon cancer patients from the UNITED study
Source: ESMO Open. 2025 Dec 30;11(1):105934. doi: 10.1016/j.esmoop.2025.105934 (PMC12804037; doi:10.1016/j.esmoop.2025.105934)
Supplement: Supplementary Material 1 [file mmc8.docx]

Supplementary material

[Supplementary Table 1: 2](#_Toc204865825)

[Supplementary Table 2 3](#_Toc204865826)

[Supplementary Figure 1 4](#_Toc204865827)

[Supplementary Figure 2 5](#_Toc204865828)

[Supplementary Figure 3 6](#_Toc204865829)

[Supplementary Figure 4 7](#_Toc204865830)

[Supplementary Figure 5 8](#_Toc204865831)

[Supplementary Figure 6 9](#_Toc204865832)

[Supplementary Figure 7 10](#_Toc204865833)

[Supplementary Figure 8 11](#_Toc204865834)

Supplementary Table 1: Determination of the automated TSR cut-off value based on prognostic value for overall survival and disease-free survival, showing 80% as the optimal cut-off.

| **Automated stromal percentage** | **OS** | **DFS** |
| --- | --- | --- |
| ≤ 40 | 4.671 | 3.320 |
| > 40 | 4.513 | 5.207 |
|  | p = 0.707**^*^**  HR = 1.246 (0.395 – 3.937)^#^ | p = .00351**^*^**  HR = 1.367 (0.699 – 2.673)^#^ |
| < 45 | 4.649 | 4.524 |
| ≥ 45 | 4.512 | 5.199 |
|  | p = 0.897**^*^**  HR = 1.220 (.448 – 3.319)^#^ | p = .232**^*^**  HR = 1.634 (.724 – 3.688)^#^ |
| ≤ 50 | 4.718 | 4.489 |
| > 50 | 4.503 | 5.185 |
|  | p = 0.285**^*^**  HR = 2.555 (1.039 – 6.282)^#^ | p = 0.171**^*^**  HR = 1.556 (0.823 – 2.944)^#^ |
| < 55 | 4.809 | 4.648 |
| ≥ 55 | 4.483 | 5.135 |
|  | p = 0.034**^*^**  HR = 0.961 (0.634 – 1.457)^#^ | p = 0.007**^*^**  HR = 2.252 (1.224 – 4.144)^#^ |
| ≤ 60 | 4.793 | 4.563 |
| > 60 | 4.469 | 5.113 |
|  | p = 0.017**^*^**  HR = 2.357 (1.144 – 4.859)^#^ | p = 0.007**^*^**  HR = 1.910 (1.188 – 3.072)^#^ |
| < 65 | 4.644 | 4.609 |
| ≥ 65 | 4.476 | 5.152 |
|  | p = 0.113**^*^**  HR = 1.495 (0.907 – 2.646)^#^ | p = 0.042**^*^**  HR = 1.444 (1.011 – 2.063)^#^ |
| ≤ 70 | 4.639 | 5.187 |
| > 70 | 4.441 | 5.051 |
|  | p = 0.032**^*^**  HR = 1.597 (1.037 – 2.458)^#^ | p = 0.023**^*^**  HR = 1.429 (1.049 – 1.946)^#^ |
| ≥ 75 | 4.627 | 5.174 |
| < 75 | 4.380 | 4.870 |
|  | p = 0.006**^*^**  HR = 1.719 (1.160 – 2.548)^#^ | p = 0.006**^*^**  HR = 1.499 (1.124 – 1.999)^#^ |
| ≤ 80 | 4.594 | 5.150 |
| > 80 | 4.299 | 4.562 |
|  | p = 0.003**^*^**  HR = 1.834 (1.222 – 2.751)^#^ | p <0.001**^*^**  HR =1.785 (1.316 – 2.419)^#^ |

**^*^**Calculated using Kaplan Meier analysis

^#^Calculated using Cox regression

OS = overall survival, DFS = disease-free survival

Supplementary Table 2: Multivariate analysis of prognostic value of TSR on disease-free survival.

| **Variables** | **p-value** | **HR** | **95% CI for HR** |
| --- | --- | --- | --- |
| TSR | 0.016 | 1.45 | 1.07 – 1.95 |
| Male gender | 0.147 | 1.25 | 0.92 – 1.69 |
| ≥75 years | 0.436 | 1.45 | 0.814 – 1.69 |
| Biopsy taken | <0.001 | 2.44 | 1.61 – 3.71 |
| Right-sided tumor | 0.889 | 1.02 | 0.75 – 1.39 |
| pT4-stage | <0.001 | 1.84 | 1.32 – 2.57 |
| pN1-stage | 0.001 | 1.95 | 1.31 – 2.91 |
| pN2-stage | <0.001 | 2.80 | 1.77 – 4.42 |
| Pathology risk factors present^a^ | 0.003 | 1.71 | 1.20 – 2.42 |
| >12 LN sampled | 0.279 | 1.29 | 0.82 – 2.03 |
| Poor differentiation^b^ | 0.891 | 0.97 | 0.61 – 1.55 |
| Adenocarcinoma | 0.299 | 0.76 | 0.47 – 1.27 |
| ACT received | 0.001 | 0.55 | 0.38 - 0.80 |

HR = hazard rate; CI = confidence interval; TSR = tumor-stroma ratio; LN = lymph nodes; ACT = adjuvant chemotherapy.

^a^Pathology risk factors are stated in Table 2. Presence of a risk factor is defined as at least one of registered risk factors. Absence is the absence of registered risk factors, as not all risk factors are registered.

^b^Differentiation grade is variously registered as separate or combined subgroups; this is then categorized into combined grades, i.e. well – moderate or poor – undifferentiated.

Supplementary Figure 1: Receiver Operating Characteristic curve stromal percentage

Receiver Operating Characteristic (ROC) curve for stromal percentage in a random sample of the UNITED cohort as determined by algorithm for disease-free survival in figure 2A and for overall survival in figure 2B. The curve illustrates discriminatory ability of stromal percentage to distinguish between outcome groups with a calculated area under the curve of 0.602 for disease-free survival and 0.600 for overall survival. The optimal cut-off value was determined using the Youden Index, calculated as J = sensitivity + specificity – 1. This analysis yielded a cut-off value of 76.5% stroma for DFS and OS, which was rounded to 77%, above which tumors were classified as stroma-high.


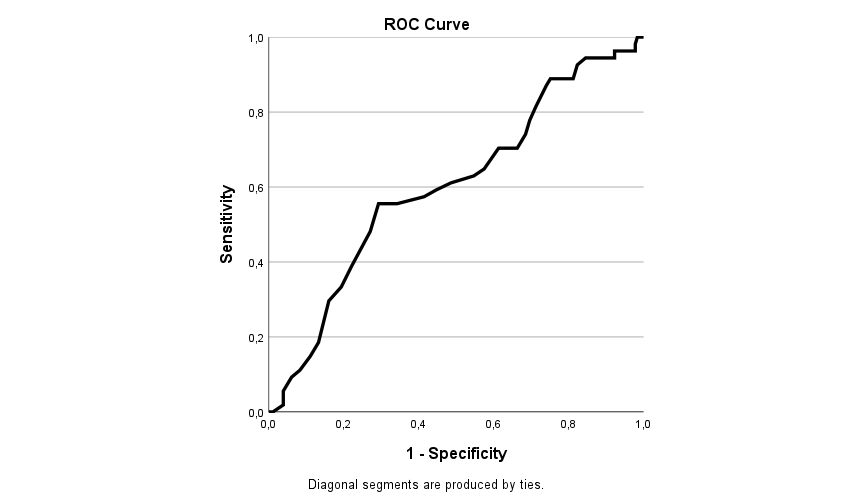


B

A


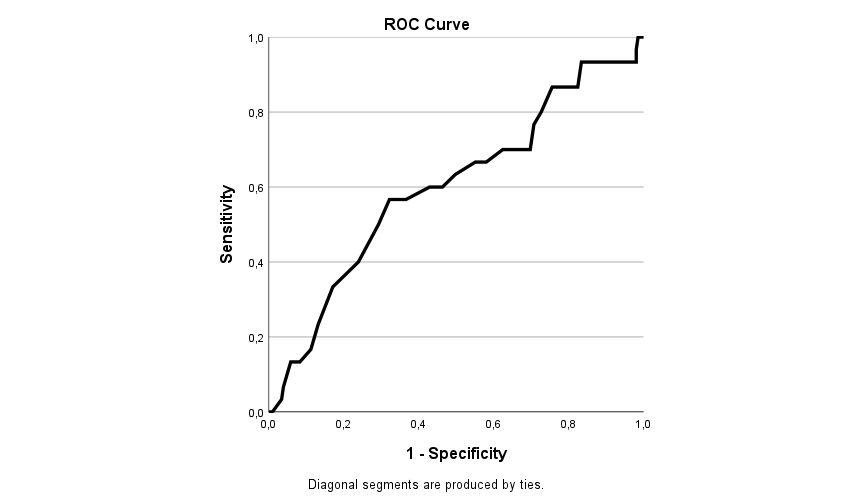


AUC = 0.602

AUC = 0.600


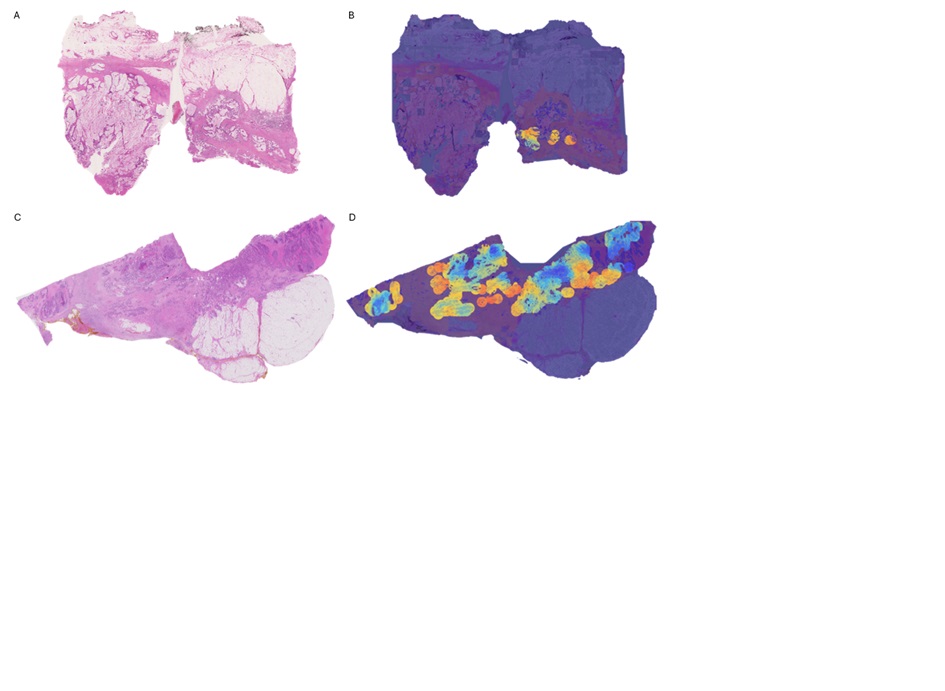
Supplementary Figure 2: Representative examples of stroma-high and stroma-low tumors analyzed by automated TSR assessment algorithm.

**A** H&E-stained section of the primary colorectal cancer. Visually assessed TSR scored as low by a trained pathologist with stromal percentage of 40%. **B** Automated TSR scored as low by algorithm with stromal percentage of 27%. **C** H&E-stained section of the primary colorectal cancer. Visually assessed TSR scored as high by a trained pathologist with stromal percentage of 80%. **D** Automated TSR scored as high by algorithm with stromal percentage of 81%.


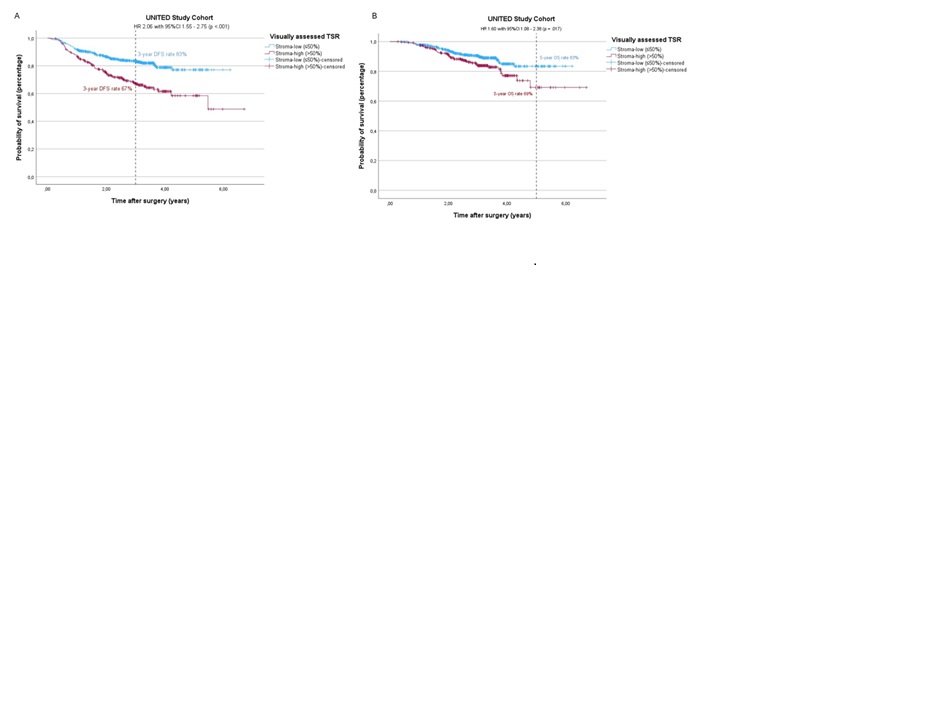
Supplementary Figure 3: Survival effect of visually assessed TSR in UNITED cohort.

**A** Disease-free survival effect of visually assessed TSR in UNITED cohort. Kaplan Meier analysis and log-rank test showing worse 3-year disease-free survival rates for visually assessed stroma-high patients (3-year DFS 67% and 83%, respectively; HR 2.06 with 95% CI 1.55 – 2.75; p <0.001). **B** Overall survival effect of visually assessed TSR in UNITED cohort. Kaplan Meier analysis and log-rank test showing worse 5-year overall survival rates for visually assessed stroma-high patients (5-year OS 69% and 83%, respectively; HR 1.60 with 95% CI 1.08 – 2.38; p <0.017).


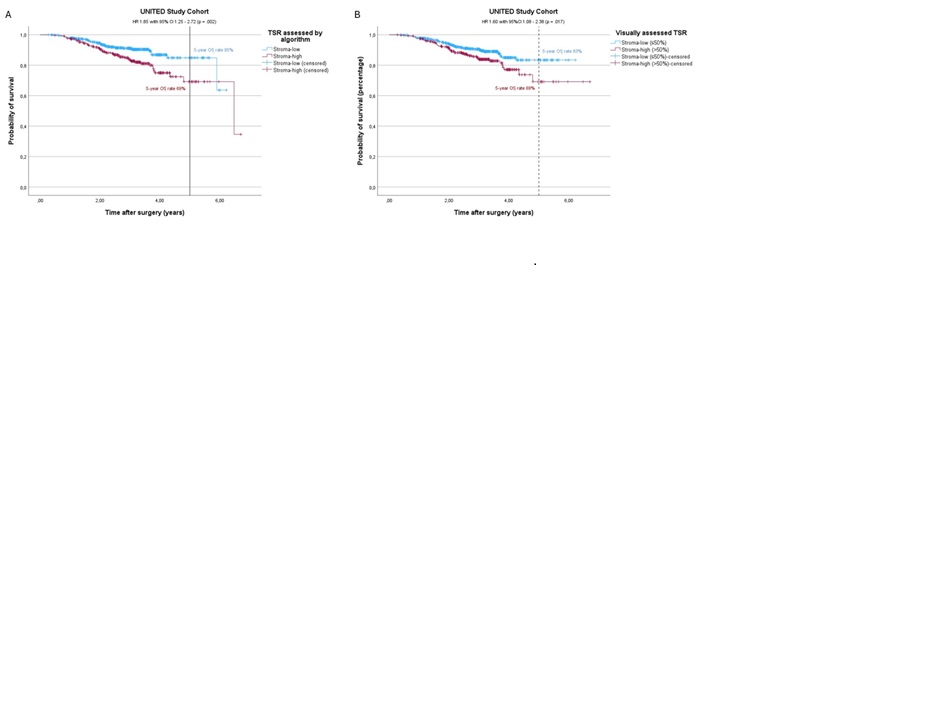
Supplementary Figure 4: Difference in overall survival between visual and automated TSR in UNITED cohort.

**A** Kaplan Meier analysis and log-rank test showing worse 5-year overall survival rates for automatically assessed stroma-high patients (5-year OS 69% and 85%, respectively; HR 1.85 with 95% CI 1.25 – 2.27; p = 0.002). **B** Kaplan Meier analysis and log-rank test showing worse 5-year overall survival rates for visually assessed stroma-high patients (5-year OS 69% and 83%, respectively; HR 1.60 with 95% CI 1.08 – 2.38; p = 0.017).


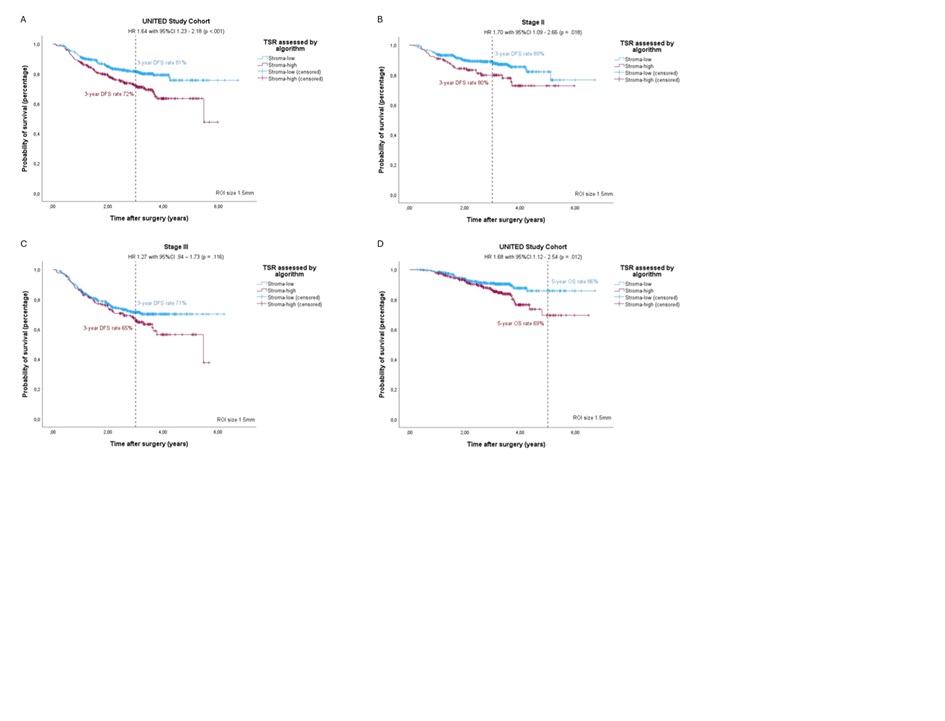
Supplementary Figure 5: Survival effect of TSR in UNITED cohort and subgroup analyses using ROI size 1.5mm.

**A** Kaplan Meier analysis and log-rank test showing worse 3-year disease-free survival rates for stroma-high patients (3-year DFS 72% and 81%, respectively; HR 1.64 with 95% CI 1.23 – 2.18; p <0.001). **B** Kaplan Meier analysis and log-rank test showing worse 3-year disease-free survival rates for stage II stroma-high patients (3-year DFS 80% and 89%, respectively; HR 1.70 with 95% CI 1.09 – 2.66; p = 0.018). **C** Kaplan Meier analysis and log-rank test in stage III patients, showing no statistically significant difference in 3-year disease-free survival rates (3-year DFS 65% and 71%, respectively; HR 1.27 with 95% CI .94 – 1.73; p <0.116) **D** Kaplan Meier analysis and log-rank test showing worse 5-year overall survival rates for stroma-high patients (5-year OS 69% and 86%, respectively; HR 1.68 with 95% CI 1.12 – 2.54; p = 0.012).


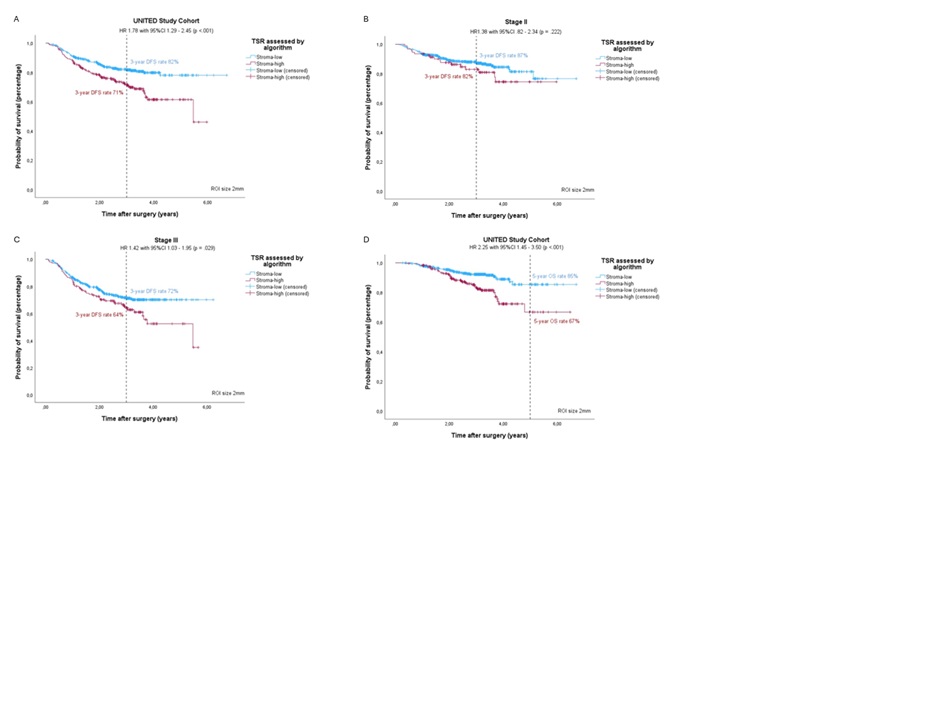
Supplementary Figure 6: Survival effect of TSR in UNITED cohort and subgroup analyses using ROI size 2mm.

**A** Kaplan Meier analysis and log-rank test showing worse 3-year disease-free survival rates for stroma-high patients (3-year DFS 71% and 82%, respectively; HR 1.78 with 95% CI 1.29 – 2.45; p <0.001). **B** Kaplan Meier analysis and log-rank test showing no statistically significant difference in 3-year disease-free survival rates (3-year DFS 82% and 87%, respectively; HR 1.38 with 95% CI .82 – 2.34; p = 0.222). **C** Kaplan Meier analysis and log-rank test in stage III patients showing worse 3-year disease-free survival rates for stroma-high patients (3-year DFS 64% and 72%, respectively; HR 1.42 with 95% CI 1.03 – 1.95; p <0.029) **D** Kaplan Meier analysis and log-rank test showing worse 5-year overall survival rates for stroma-high patients (5-year OS 67% and 85%, respectively; HR 2.25 with 95% CI 1.45 – 3.50; p <0.001).


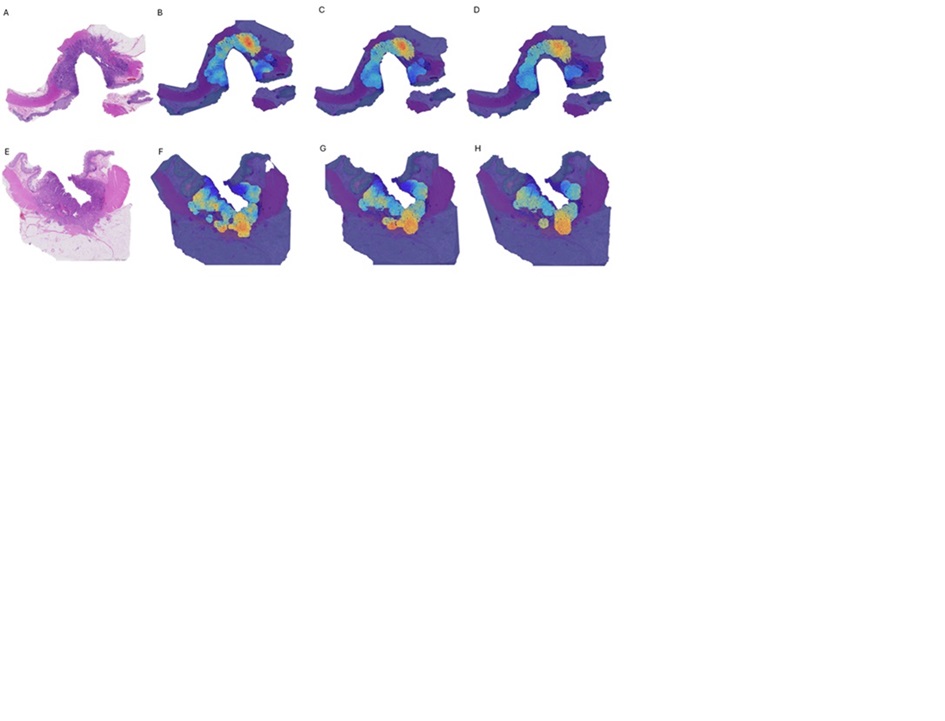
Supplementary Figure 7: Difference in automated TSR assessment between different ROI sizes.

**A** H&E-stained section of the primary colorectal cancer. **B** Visual output of the TSR algorithm with ROI size 1 mm with highest stromal percentage of 81%, classifying as TSR high. **C** Visual output of the TSR algorithm with ROI size 1.5 mm with highest stromal percentage of 82%, classifying as TSR high. **D** Visual output of the TSR algorithm with ROI size 2 mm with highest stromal percentage of 77 %, classifying as TSR high. **E** H&E-stained section of the primary colorectal cancer. **F** Visual output of the TSR algorithm with ROI size 1 mm with highest stromal percentage of 77%, classifying as TSR high. **G** Visual output of the TSR algorithm with ROI size 1.5 mm with highest stromal percentage of 79%, classifying as TSR high. **H** Visual output of the TSR algorithm with ROI size 2 mm with highest stromal percentage of 73%, classifying as TSR low.


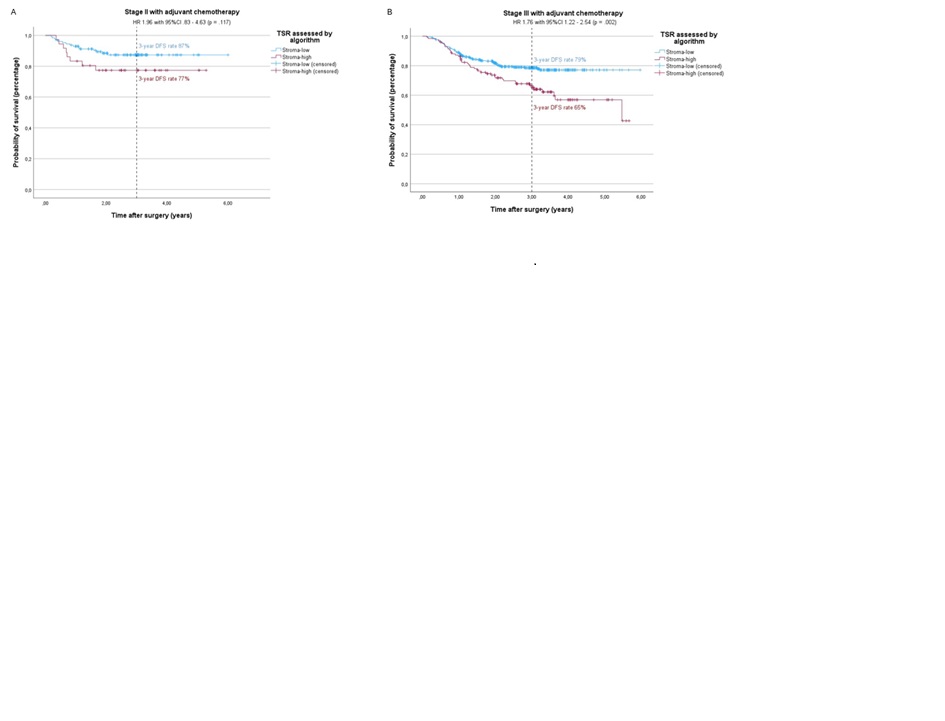
Supplementary Figure 8: Survival effect of automated TSR in stage II and III patients with adjuvant chemotherapy without stratification for age.

**A** Kaplan Meier analysis and log-rank test showing in stage II patients receiving adjuvant chemotherapy, showing no statistically significant difference in 3-year disease-free survival rates (3-year DFS 77% and 87%, respectively; HR 1.96 with 95% CI .83 – 4.63; p = 0.117). **B** Kaplan Meier analysis and log-rank test in stage III patients receiving adjuvant chemotherapy, showing worse 3-year disease-free survival rates for stroma-high patients despite treatment (3-year DFS 65% and 79%, respectively; HR 1.76 with 95% CI 1.22 – 2.54; p = 0.002).
