# Supplementary material for: Automating tumor–stroma ratio quantification in colon cancer patients from the UNITED study
Source: ESMO Open. 2025 Dec 30;11(1):105934. doi: 10.1016/j.esmoop.2025.105934 (PMC12804037; doi:10.1016/j.esmoop.2025.105934)
Supplement: Supplementary Material 2 [file mmc9.docx]

**Glossary**

| ACT = adjuvant chemotherapy |
| --- |
| AI = artificial intelligence |
| AJCC = American Joint Committee on Cancer |
| CI = confidence interval |
| CRC = colorectal cancer |
| DFS = disease-free survival |
| H&E = hematoxylin-eosin |
| ICCR = International Collaboration on Cancer Reporting |
| MREC = Medical Research Ethics Committee |
| OS = overall survival |
| PFS = progression-free survival |
| ROC = Receiver Operating Characteristic |
| ROI = region of interest |
| TNM = tumor node metastasis |
| TSR = tumor-stroma ratio |
| UICC = Union for International Cancer Control |
| UNITED study = Uniform Noting for International application of Tumor-stroma ratio as Easy Diagnostic tool |
| WSI = whole slide image |
